# NOP agonist AT-403 promoted sleep in lactic acid-induced acute pain model

**DOI:** 10.3389/fpain.2025.1659121

**Published:** 2025-09-22

**Authors:** Bethany E. Pierce, Harlie A. McKelvey, Mary H. Hite, John M. Lyerly, Ivan M. Krizan, Kimberly M. Holter, Rong Chen, Nurulain T. Zaveri, Robert W. Gould

**Affiliations:** ^1^ Department of Translational Neuroscience, Wake Forest University School of Medicine, Winston-Salem, NC, United States; ^2^ Astraea Therapeutics, Mountain View, CA, United States

**Keywords:** pain, sleep, analgesics, opioid, mu opioid receptor agonist, nociception opioid receptor agonist, lactic acid, pain-depressed behavior

## Abstract

The majority of patients with acute pain experience sleep disturbances that persist despite analgesic treatments such as mu opioid receptor (MOP) agonists and non-steroidal anti-inflammatory drugs (NSAIDs). Further, sleep disturbances increase pain sensitivity, demonstrating a bi-directional relationship between pain and sleep. Given that commonly prescribed MOP agonists disrupt sleep in pain-naïve subjects, it is possible that analgesics exacerbate sleep disturbances associated with pain states. Thus, pain-induced sleep disturbances remain an understudied and undertreated symptom impacting overall quality of life for which development of novel analgesics is critical. Nociceptin/Orphanin FQ opioid receptor (NOP) agonists have shown promise as a novel class of analgesic, and, given sleep-promoting effects in naïve subjects, may improve pain-induced sleep disturbances. We examined the effects of intraperitoneal lactic acid administration, a noxious stimulus which produces acute abdominal pain, on sleep alone and in the presence of analgesics morphine (MOP agonist), meloxicam (NSAID), and novel NOP agonist AT-403. Male and female Sprague Dawley rats were implanted with wireless electroencephalography (EEG) devices to assess sleep duration and brain function using quantitative EEG analyses. Lactic acid dose-dependently decreased rapid eye movement (REM) and non-REM (NREM) sleep duration, and, consistent with prior studies, increased stretching and decreased rearing and grooming behaviors in a concentration-dependent manner. Morphine significantly decreased NREM and REM sleep in pain-naïve states and did not improve sleep following lactic acid administration. Additionally, lower doses of morphine increased high frequency power spectra. In contrast, meloxicam did not affect sleep or quantitative EEG in pain-naïve rats, nor alter lactic-acid induced effects. AT-403 increased NREM sleep duration and slow wave activity during NREM sleep, decreased NREM sleep latency and REM sleep duration both alone and in the presence of lactic acid; at the higher doses tested, AT-403 shifted relative spectral distribution from higher to lower frequency ranges, indicative of a sedative effect. In contrast, AT-403 attenuated lactic acid-induced behaviors and promoted sleep at doses that did not decrease locomotor function. Together, these data demonstrate that current analgesics do not sufficiently alleviate acute pain-induced sleep disturbances whereas NOP agonists represent a novel mechanism for the potential treatment of pain-induced sleep disturbances.

## Introduction

Sleep disturbances are common complaints associated with acute pain (i.e., pain following direct injury or painful stimulus), with pain severity corresponding with worse sleep (1–10). Subjects with moderate acute pain resulting from neck or back pain, ankle sprain, or excessive exercise report having difficulty falling asleep (increased sleep latency), sleep fragmentation, and next-day fatigue (11–14). In more intense acute pain states following surgery, 23%–62% of elective surgical patients experienced severe sleep disturbances that persisted for 4 days or longer (10, 15, 16). As sleep disruptions increase pain perception and sensitivity (2, 6, 17–19) and impact post-surgical recovery duration (2, 6, 17, 19), alleviating sleep disturbances could improve pain-related symptoms. Moreover, these sleep disturbances can cause downstream reductions in mood, cognitive ability, and quality of life (20–26). However, the majority of subjects in the above studies received analgesics such as mu opioid receptor (MOP) agonists, non-steroidal anti-inflammatory drugs (NSAIDs), NMDA receptor antagonists, or gabapentin, many of which disrupt sleep in a pain-naïve setting (27–31) making it difficult to differentiate pain-related sleep disturbances from drug-related sleep disturbances.

First-line treatments for acute pain include MOP agonists and NSAIDs. Despite high efficacy for moderate to severe pain, MOP agonists are associated with numerous side effects including high abuse potential, respiratory depression, and sleep disturbances (32–38). Acute administration of MOP full and partial agonists such as morphine, heroin, remifentanil, methadone, and buprenorphine increase sleep latency (time until sleep onset), suppress rapid eye movement (REM) sleep and non-REM (NREM) sleep, and reduce sleep quality in healthy animals (34, 35, 36–38) and humans (39–42). NSAIDs are widely used to treat mild to moderate pain and have minimal side effects, but are not as efficacious as MOP agonists for severe pain (43–46). Some clinical literature suggests that NSAIDs mildly increase time awake and nocturnal awakenings while others report no significant alterations in objective or subjective sleep measures (47, 48). Although post-operative sleep disturbances are present for the majority of patients, prescriptions for sleep medications are relatively rare, with only 3% of patients being prescribed with a benzodiazepine drug (16, 49). Despite sleep promotion (48, 50), benzodiazepines induce light stage sleep while inhibiting deep, restorative slow wave sleep (50, 51). In addition, benzodiazepines have significant abuse liability when taken long term (49, 52, 53), and can increase the respiratory depressive effects of opioids, increasing the risk of fatal overdose (53, 54). Thus, there is a need for high efficacy analgesics that either promote sleep, or at minimum, are absent of sleep disturbances and are devoid of adverse side effects.

It is difficult to differentiate the direct pharmacological effects of analgesics (e.g., analgesic or sleep-altering effects) from the indirect effects (e.g., alleviating pain to impact sleep) in a clinical setting. Polysomnography and the use of electroencephalography (EEG) to identify/quantify sleep stages and measure brain activity is the gold standard for sleep studies that can be applied across species. However, many clinical studies use subjective sleep measures, often retrospectively, despite a frequent disconnect between these measures and polysomnographic assessments (55–57). Thus, rigorous clinical and preclinical studies are needed to understand both the direct effects of pain on sleep alone and the pharmacological effects of analgesics on sleep in a pain-naïve vs. pain state in order to identify novel analgesics that effectively minimize pain directly and indirectly through sleep-promoting mechanisms.

Nociceptin/orphanin FQ opioid receptor (NOP) agonists are a promising therapeutic approach for both acute and chronic pain, possessing both a favorable analgesic and side effect profile. Similar to classic MOP agonists, NOP agonists reduce stimulus-evoked nociceptive responses in healthy animals (58–60) and demonstrate analgesic efficacy in acute (61–64) and chronic pain (61, 65, 66) models. NOP agonists exert their analgesic effects primarily through peripheral inhibition of pain signals in the dorsal root ganglion (DRG) of the spinal cord, and activation of ascending and descending pain pathways in the periaqueductal gray (PAG) (62, 67, 68). However, unlike MOP agonists which suppress NREM and REM sleep, NOP agonists increase NREM sleep, decrease sleep latency, and improve sleep maintenance in a pain naïve state in both humans and rats (69–71). Due to their dual analgesic and sleep-promoting effects, we hypothesized that NOP agonists would prevent pain-induced sleep disturbances.

Although several preclinical rodent studies have shown that acute pain increases sleep latency and decreases sleep duration across a variety of acute and chronic pain models (72–77), few studies have examined the effects of analgesics on sleep in acute or chronic pain models (78). Lactic acid (LA) is a mildly noxious stimulus that reliably produces pain-associated behaviors (e.g., stretching and grooming) and, at higher concentrations, pain-depressed behaviors (e.g., decreased exploratory behaviors such as rearing) (79–82). First, we examined the effects of intraperitoneal (i.p.) administration of lactic acid on sleep and oscillatory brain activity and hypothesized that this acute pain model would produce sleep disruptions. Second, we examined the effects of morphine (MOP agonist), meloxicam (NSAID), and AT-403 [a full agonist at the NOP receptor (83)] on sleep/wake duration alone and in combination with lactic acid. As hypothesized, we found that morphine disrupted sleep and did not alleviate lactic acid-induced sleep disturbance whereas the NOP agonist AT-403 promoted sleep regardless of lactic acid treatment, demonstrating potential clinical utility for pain-induced sleep disturbances.

## Methods

### Subjects

Sprague-Dawley rats (35 males, 20 females; Envigo, Indianapolis, IN, USA) were individually (EEG experiments) or pair housed (behavior alone experiments) in opaque cages (8 in × 10 in × 8 in). All rats were 2 months old at the beginning of the study and maintained on a 12-hour light/12-hour dark cycle in a temperature and humidity-controlled room (65°F–75°F, 20%–40% humidity) with *ad libitum* access to food and water. All behavioral testing occurred in the first 4 h of the light cycle. See supplemental files for information about specific animal numbers in each experiment. All animal care procedures were approved by the Wake Forest University Animal Care and Use Committee and adhered to the guidelines set forth in the National Institutes of Health's *Guide for the Care and Use of Laboratory Animals*.

### Drugs

Morphine [(−) morphine sulfate pentahydrate; Sigma-Aldrich and NIDA Drug Supply Program; 0.1–10 mg/kg, i.p.] and meloxicam [Patterson Veterinary Supply; 0.5–2 mg/kg, subcutaneous (s.c.)] were dissolved or diluted in sterile saline, respectively. AT-403 (67) (0.003–0.3 mg/kg, s.c.), synthesized as previously described and provided by Astraea Therapeutics was dissolved in a 20% beta-cyclodextrin/sterile deionized water solution. Lactic acid (Sigma-Aldrich; 1.0%–5.6%, i.p.) was diluted in sterile deionized water. All drugs were administered at a volume of 1 mL/kg Testing days were separated by a minimum of 3 days and dose/concentration order within each compound was counterbalanced. Effects of a single compound were tested prior to switching to different compounds.

### Lactic acid treatment

#### Experiment 1a: lactic acid-induced acute abdominal pain

Two-month old male (*n* = 12) and female (*n* = 12) Sprague Dawley rats were placed individually in clean, bedding-free cages identical to their home cage for a 30-minute habituation period. Then, the animals were administered lactic acid (0%, 1%, 1.8%, 3.2%, or 5.6%, i.p.; testing days were separated by 3 days and doses were counterbalanced for full within-subjects analysis) and their behavior was recorded by cameras positioned above the cages for 30 min. Animals were left undisturbed by experimenters during behavioral recording. Frequency of stretching, rearing, and grooming behavior was manually recorded by a trained observer blinded to the administered concentration. In line with previous studies, we found that effects of i.p. lactic acid administration remain stable over repeated administration (84–87).

#### Experiment 1b

Rats (*n* = 7, 5 month old male rats) were administered morphine (vehicle, 0.1, or 1.0 mg/kg; i.p.), meloxicam (vehicle, 2.0 mg/kg; s.c.), or AT-403 (vehicle, 0.003, 0.01, 0.03, or 0.1 mg/kg; s.c.) 1 h prior to 5.6% lactic acid administration. Frequency of stretching, rearing, and grooming was determined to assess anti-nociceptive activity. The lactic acid concentration was based on EEG studies that showed that only 5.6% lactic acid produced measurable sleep disruptions (see results). A one-way ANOVA with repeated measures or dependent *t*-tests were used to analyze the sum of stretching, rearing, and grooming behavior.

### Electroencephalography (EEG)

#### EEG surgery

Rats were anesthetized using isoflurane gas (3%–5% for induction, 1%–3% maintenance) and two incisions were made: one in the animal's dorsal flank and another at the skull. The telemetric transmitter and battery pack (HD-S02, Data Sciences International, Minneapolis, MN) were placed subcutaneously along the flank and four wires were tunneled to the skull as previously described (88–90). Two wires were placed anti-parallel and secured to the nuchal muscle for electromyography (EMG) recordings. Then, two 1.2 mm holes were drilled in the animal's skull and placed above the frontal (+2 mm anterior to Bregma and +2 mm from the midline) and contralateral occipital lobe (−6 mm posterior to Bregma and −2 mm from the midline). Wires for EEG recordings were looped and placed into the holes in contact with the dura, and secured with dental cement. All incisions were closed with dissolvable sutures. Rats received meloxicam (1 mg/kg s.c.; once per day) for 2 days and Baytril (antibiotic; 5 mg/kg s.c.; once per day) for 5 days post-surgery. Rats recovered for a minimum of 1 week prior to experimental testing. All EEG recordings occurred in freely-moving animals from their home cage, and EEG, EMG, and homecage locomotor activity counts were transmitted telemetrically to a receiver beneath each rat's home cage. All EEG recordings were initiated at the beginning of the light cycle (Zeitgeber Time; ZT) 0) and lasted for 24 h.

#### Experiment 2: effects of lactic acid on sleep

Following a 2-hour baseline EEG recording (ZT 0–2), rats (*n* = 11 male, 8 female) were administered lactic acid (vehicle, 1%, 1.8%, 3.2%, or 5.6% i.p.) at ZT2; concentration order was randomized. EEG data were analyzed during the first 6 h post lactic acid administration due to transient effects that persisted for only 1–2 h. Due to more prominent effects in males than in females, subsequent lactic acid studies only included males.

#### Experiment 3: effects of analgesics on sleep

Following a 2-hour baseline recording (ZT 0–2), rats were administered morphine (*n* = 8; vehicle, 0.1, 1.0, 3.0, or 10.0 mg/kg, i.p.), meloxicam (*n* = 9; vehicle, 0.5, 1.0, or 2.0 mg/kg, s.c.), or AT-403 (*n* = 15; vehicle, 0.01, 0.03, 0.10, 0.30 mg/kg; s.c.) at ZT2. EEG data were recorded and analyzed for a full 24-hour period; data are reported in 1-hour bins to examine the time-dependent effects or cumulative duration of the 3 h following administration.

#### Experiment 4: effects of analgesics + lactic acid on sleep

Following a 1-hour baseline recording (ZT 0–1), rats were administered morphine (*n* = 9; vehicle, 0.1 or 1 mg/kg, i.p.), meloxicam (*n* = 9; vehicle or 2 mg/kg s.c.), or AT-403 (*n* = 8; vehicle, 0.003, 0.01, 0.03, or 0.10 mg/kg; s.c.) at ZT 1 and lactic acid (5.6%, i.p.) at ZT 2. Though EEG was recorded for a full 24-hour cycle, data were only analyzed during the first 6 h post lactic acid administration due to transient effects of lactic acid (see Results).

Lastly, to examine the relationship between sleep and pain-related behaviors in combination with analgesics, we conducted video recordings from a subset of the animals that underwent the analgesics + lactic acid EEG recordings (*n* = 7, 5-month-old male rats). Following a 30-minute habituation period, meloxicam (2.0 mg/kg; s.c.), morphine (0.1 or 1.0 mg/kg; i.p.), AT-403 (0.003 mg/kg; s.c.), or vehicle were administered 1 h prior to lactic acid (5.6%, i.p.). Frequency of stretching, rearing, and grooming were determined. Within-subject linear regressions were used to examine correlations between sleep duration (NREM sleep within the first 60 min post-administration; REM sleep within the first 3 h) and stretching behavior (within the first 30 min) following lactic acid administration.

#### Experiment 5: effects of AT-403 on locomotor activity

To determine whether AT-403 affected locomotor function that could confound interpretation of lactic acid-induced behaviors, we assessed locomotor coordination and balance using the Rotarod task (Med Associates, ENV-571R). Animals (*n* = 8 males, 8 females) were first trained for 5 sessions before AT-403 testing. Rats were placed on a grooved beam (7 cm diameter, 11.5 cm wide) elevated 43 cm above the ground that rotated at an escalating speed (4, 8, 12, 16, 20, 24, 28, 32, 36, 40 rotations per minute, 30 s each). Trials lasted for a total of 5 min or until the rat fell off. On test days, AT-403 (vehicle, 0.003, 0.01, 0.03, or 0.1 mg/kg) was administered 1 h prior to the test. The trial duration and maximum rotations per minute (RPM, speed) were recorded (Rotarod 2 SOF-571) and mixed-effects one-way ANOVAs followed by Dunnett's *post hoc* tests were used to determine dose effects.

### Data collection and analysis

EEG, EMG, and locomotor activity were collected using Ponemah Software version 6.5 (DSI) as previously described (88–90) with a continuous sampling rate of 500 Hz. Trained observers, blinded to condition, scored each 10-second epoch as wake, REM sleep, NREM sleep, or artifact based on standardized EEG characteristics using Neuroscore software (DSI). Duration of time in each state (NREM and REM sleep; wake) was summed in 1, 3, and 6 h bins following compound administration. Sleep continuity/fragmentation was examined by quantifying the (1) number of brief arousals (defined as 20–30 s waking durations occurring in the middle of a NREM sleep bout), (2) average length/duration and (3) frequency of NREM sleep bouts or REM sleep bouts occurring specifically within the first 3 h following compound administration, using custom MATLAB scripts. Additionally, sleep latency was determined, defined as time to first NREM sleep bout (≥30 s) or REM sleep bout (≥20 s) after compound administration. Subsequently, spectral power distribution was calculated in 1 Hz bins from 0.5 to 100 Hz using a Fast Fourier Transform with a Hamming window and overlap ratio of 0.5 within each 10-second epoch. Quantitative EEG (qEEG) data were quantified as the relative power (percent of total power in each epoch) within specific frequency bands (delta [0.5–4 Hz], theta [4–8 Hz], alpha [8–12 Hz], sigma [12–16 Hz], beta [16–24 Hz], low gamma, [30–50 Hz], high gamma, [50–100 Hz] and total gamma power [30–100 Hz]) analyzed within each arousal state (wake, REM sleep, and NREM sleep; artifact was excluded from analysis) using custom MATLAB scripts. Data were expressed as the % change from each individual rat's 2-hour baseline recording prior to drug administration before being averaged together as a group.

### Statistical analysis

Mixed-effects two-way ANOVAs followed by Dunnett's *post hoc* tests were used to assess concentration or dose effects of lactic acid, morphine, meloxicam, AT-403, and combinations (compound + lactic acid) on sleep/wake state duration, frequency band relative power, and homecage locomotor activity over time (30 min or 1-hour bins) compared to vehicle. Mixed-effects one-way ANOVAs followed by Dunnett's *post hoc* tests were used to assess dose effects during single timepoints (1, 3, and 6 cumulative hours post-dosing) on the same measures as well as measures of sleep continuity (bout duration, frequency, brief arousals). Paired *t*-tests were used to assess the effects of vehicle vs. 2 mg/kg meloxicam + 5.6% lactic acid. When examining sex differences, two-way ANOVAs followed by Dunnett's *post hoc* tests were used to assess dose × sex effects. Significance was always defined as *p* < 0.05.

## Results

### Lactic acid-induced acute abdominal pain alone and in combination with analgesics

**Figure 1:**
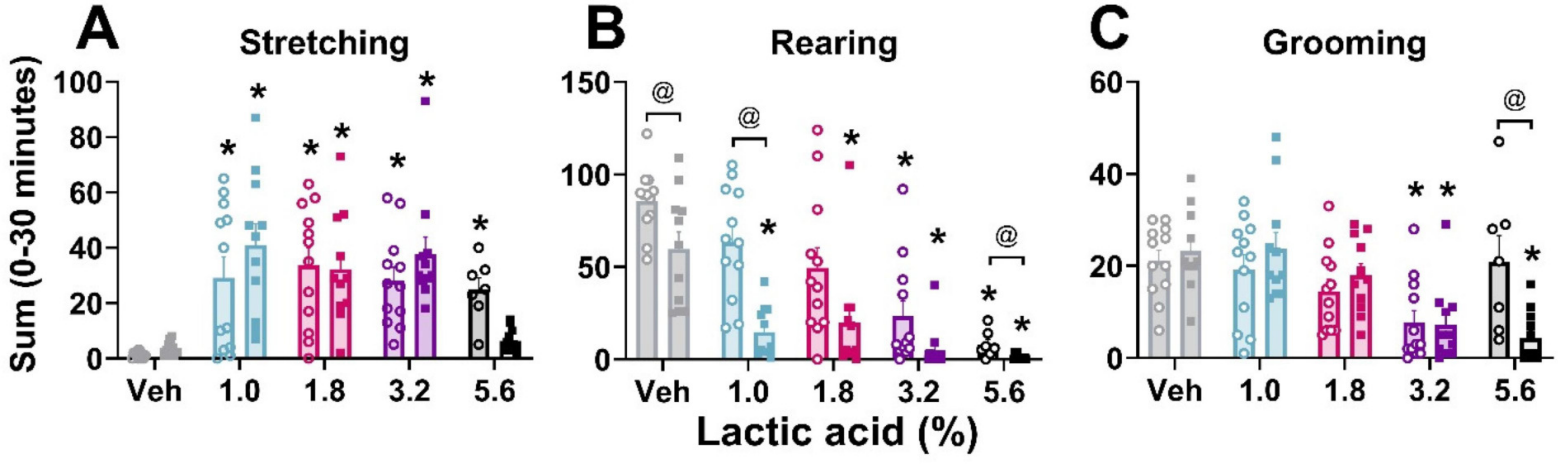
Lactic acid–induced behaviors. Lactic acid increased stretching **(A)** and decreased rearing **(B)** and grooming **(C)** behaviors in males (open circles) and females (squares) during the first 30 min post-administration. *p* < 0.05; *, concentration significantly different from respective within-sex vehicle; @, significant differences between males and females. Data expressed as the average ± SEM in error bars.

In order to verify that lactic acid influenced pain-associated behaviors, stretching, rearing, and grooming behaviors after lactic acid administration were compared to vehicle treatment for each animal. Two-way ANOVAs revealed a main effect of concentration on all measures, a main effect of sex on rearing behavior, and an interaction (concentration × sex) on grooming behavior. *Post hoc* analyses revealed increases in stretching (all tested concentrations in males; 1%–3.2% in females), and decreases in rearing (3.2% and 5.6% in males; all tested concentrations in females) and grooming (3.2% in males; 3.2% and 5.6% in females) (Table 1 and Figure 1). Sex differences were present for rearing (vehicle, 1.0%, and 5.6%) and grooming (5.6%); males exhibited more rearing and grooming behaviors than females (Table 1 and Figure 1).

**Table 1:** Effects of lactic acid on sleep and pain-related behaviors in male and female rats.

DV	Factor	DF	*F*	*p*	*	*Post hoc* results	Timepoints	*p* value
Lactic acid (%)								
Mixed effects two-way ANOVA								
Wake (1 h bins)	Concentration	3.181, 57.25	7.123	0.0003	***	1.0%	4	0.0040
	Time	4.9, 88.21	91.32	<0.0001	****	1.8%	4	0.0021
	Int	11.09, 199.7	2.670	0.0031	**	3.2%	4	0.0114
						5.6%	3, 4	0.0085, <0.0001
NREM (1 h bins)	Concentration	3.452, 62.13	2.724	0.0445	*	1.0%	4	0.0407
	Time	3.044, 54.79	51.36	<0.0001	****	1.8%	none	
	Int	10.68, 192.2	2.008	0.0310	*	3.2%	4	0.0201
						5.6%	3, 4	0.0135, 0.0007
REM (1 h bin)	Concentration	2.904, 52.27	7.432	0.0004	***	1.0%	4	0.0134
	Time	4.119, 74.15	70.81	<0.0001	****	1.8%	1, 4, 5	0.0360, 0.0042, 0.0112
	Int	10.59, 190.6	3.211	0.0006	***	3.2%	4, 5	<0.0001, 0.0008
						5.6%	3–6	0.0132, <0.0001, 0.0033, 0.0123
Wake (0–3 h)	Concentration	3.315, 56.35	13.51	<0.0001	****	Males	3.2, 5.6	0.0298, 0.0004
	Sex	1, 17	0.00188	0.9659	ns	Females	5.6	0.0229
	Int	4, 68	2.652	0.0404	ns	Sex	N/A	
NREM (0–3 h)	Concentration	3.436, 58.40	7.288	0.0002	***	Males	5.6	0.0005
	Sex	1, 17	0.008899	0.9259	ns	Females	None	
	Int	4, 68	1.915	0.1178	ns	Sex	N/A	
REM (0–3 h)	Concentration	2.989, 50.81	19.13	<0.0001	****	Males	1.8, 3.2, 5.6	0.0121, 0.0049, 0.0008
	Sex	1, 17	0.4142	0.5284	ns	Females	1, 1.8, 3.2, 5.6	0.0001, 0.0288, 0.0007, 0.0263
	Int	4, 68	2.293	0.0682	ns	Sex	N/A	
NREM latency	Concentration	2.912, 49.51	4.325	0.0093	*	Males	5.6%	0.0343
	Sex	1, 17	1.974	0.1781	ns	Females	none	
	Int	4, 68	1.329	0.2680	ns	Sex	N/A	
REM latency	Concentration	2.948, 62.65	10.52	<0.0001	****	Males	1.8, 5.6%	0.0305, 0.0015
	Sex	1, 85	2.521	0.1160	ns	Females	none	
	Int	4, 85	2.292	0.0661	ns	Sex	N/A	
Stretching (0–30 min)	Concentration	2.347, 59.27	14.61	<0.0001	****	Males	1, 1.8, 3.2, 5.6	0.0134, 0.0012, 0.0007,0.0029
	Sex	1, 101	0.05392	0.8168	ns	Females	1, 1.8, 3.2	0.0038, 0.0069, 0.0017
	Int	4, 101	2.253	0.0686	ns	Sex	N/A	
Rearing (0–30 min)	Concentration	2.549, 64.36	24.80	<0.0001	****	Males	3.2, 5.6	<0.0001, 0.0001
	Sex	1, 101	30.17	<0.0001	****	Females	1, 1.8, 3.2, 5.6	0.0050, 0.0242, 0.0015, 0.0003
	Int	4, 101	2.068	0.0905	ns	Sex	Vehicle, 1, 5.6	0.0254, 0.0001, 0.0433
Grooming (0–30 min)	Concentration	3.350, 84.60	9.607	<0.0001	****	Males	3.2	0.0018
	Sex	1, 101	0.5795	0.4483	ns	Females	3.2, 5.6	0.0112, 0.0002
	Int	4, 101	3.939	0.0052	**	Sex	5.6	0.0285

Note: timepoints refer to hours post start of recording (8 timepoints total).

ns, not significant; h, hour; min, minute; Int, interaction.

* *p* < 0.05.

** *p* < 0.01.

*** *p* < 0.001.

**** *p* < 0.0001.

### Effects of lactic acid on sleep

**Figure 2:**
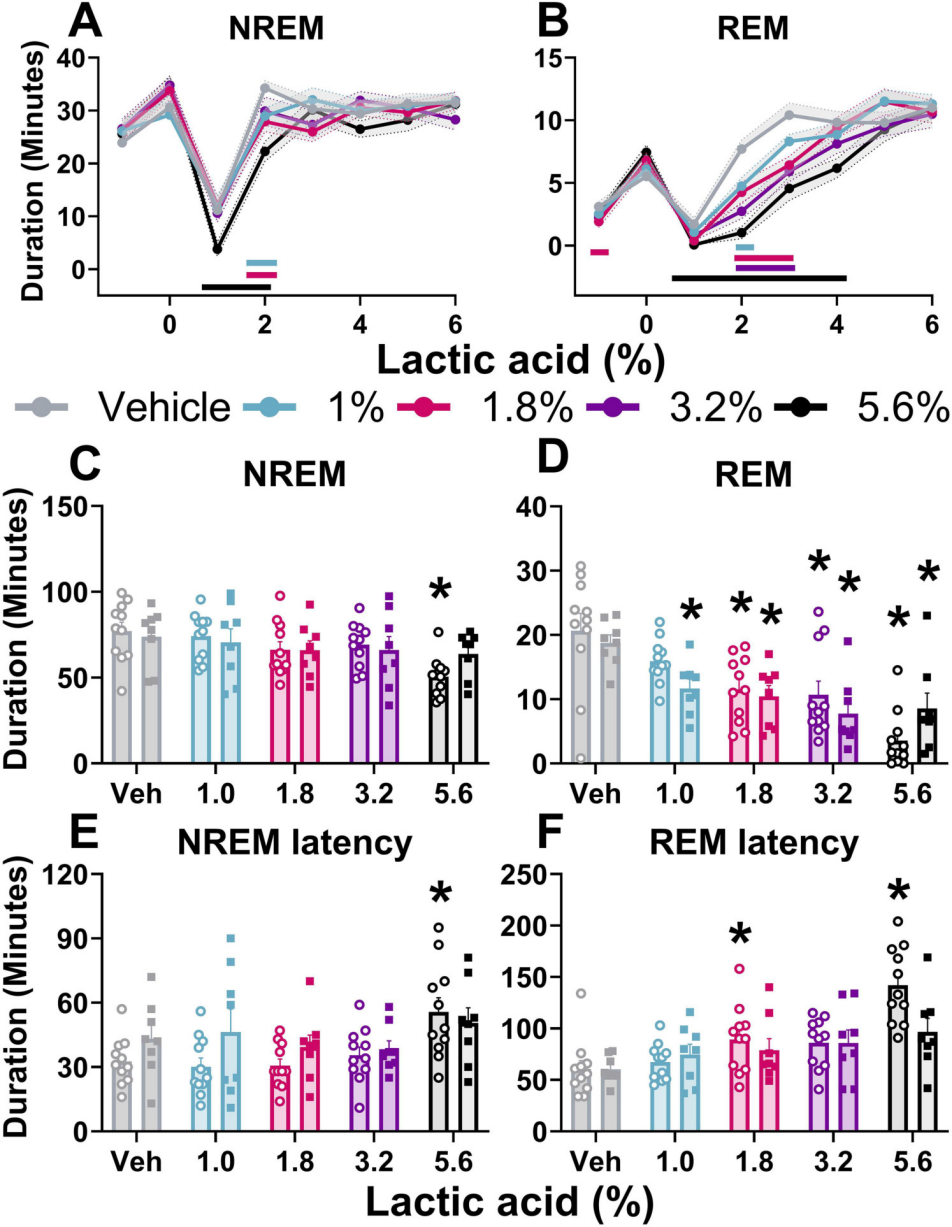
Lactic acid-induced sleep disturbances. NREM sleep and REM sleep duration is displayed in 1-hour bins for 6-hours post-dosing **(A,B)** and summed for the 3 h post-lactic acid administration **(C–F)**. Lactic acid decreased NREM sleep **(A)** and REM sleep **(B)** duration (males and females, collapsed) over time. In the summed 3 h post-dosing, lactic acid decreased NREM **(C)** and REM **(D)** sleep duration. Lactic acid also increased NREM sleep **(E)** and REM sleep **(F)** latency in males (open circles) but not in females (squares). *p* < 0.05; colored horizontal lines represent timepoints that are significant from vehicle at respective concentrations **(A,B)**; * concentration significant from respective within-sex vehicle **(C–F)**. Data expressed as the average ± SEM in error bars or shaded borders.

Two-way ANOVAs collapsed across sexes revealed that there was a main effect of lactic acid concentration on sleep/wake state (wake, NREM sleep, REM sleep) duration. *post hoc* analyses revealed that lactic acid increased time awake (1, 1.8, 3.2, 5.6; data not shown), and decreased NREM sleep (1.0%, 3.2%, 5.6%) and REM sleep (1.8%, 3.2%, 5.6%) across the time course for 1–2 h post-administration (Table 1 and Figures 2A,B). Two-way ANOVAs assessed effects of concentration and sex during the cumulative 3 h post-administration, which revealed significant concentration effects across wake, NREM sleep, REM sleep, NREM sleep latency, and REM sleep latency but there were no sex differences or interaction effects (Table 1 and Figures 2C–F). In the summed 3-hour post-administration period, lactic acid decreased NREM sleep (5.6%), decreased REM sleep (1.8%, 3.2%, and 5.6%), and increased time awake (3.2% and 5.6%; data not shown) in male rats (Table 1 and Figures 2C,D). Latency to NREM sleep (5.6%) and REM sleep (1.8% and 5.6%) were also increased in males (Table 1 and Figures 2E,F). In females, lactic acid increased wake (5.6%; data not shown), decreased REM sleep (1.0%, 1.8%, 3.2%, and 5.6%), and had no effect on NREM sleep or NREM/REM sleep latency (Table 1 and Figures 2C–F). Lastly, when male and female data were combined, lactic acid did not significantly affect number of brief arousals, NREM sleep bout sum or average duration, but decreased REM bout sum and duration (1.8%, 3.2%, 5.6%, all *p* < 0.05 compared to vehicle; Supplementary Figure S1, see Supplementary Table S1 for statistics).

### Effects of analgesics on sleep

**Figure 3:**
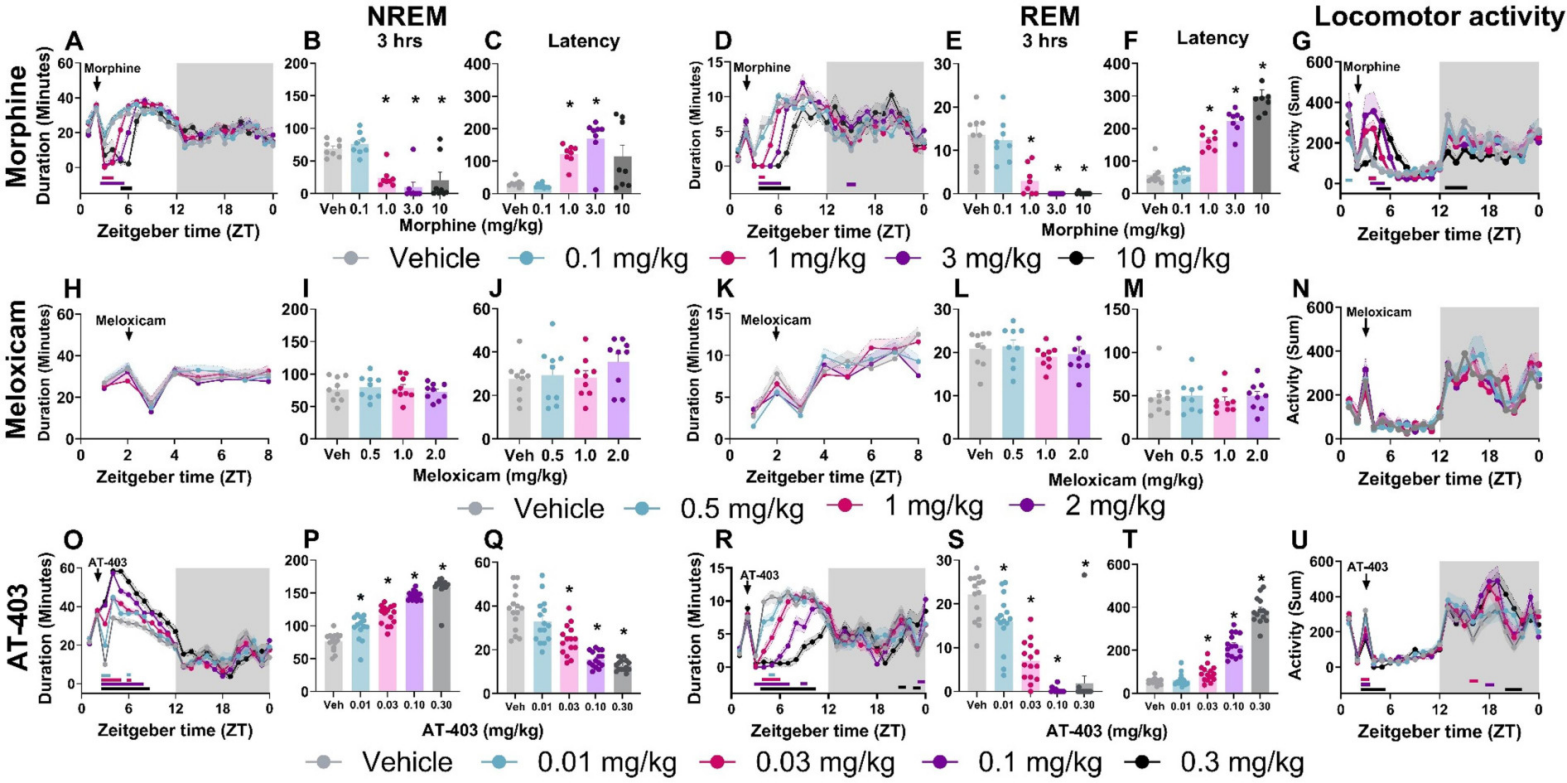
AT-403 promoted NREM sleep. NREM sleep, REM sleep duration and corresponding homecage locomotor activity displayed in both 1-hour bins before and after drug administration **(A,D,G,H,K,N,O,R,U)** and in 3-hour sums following drug administration **(B,E,I,L,P,S)**. Latency to first NREM **(C,J,Q)** or REM **(F,M,T)** sleep bout following drug administration. Morphine significantly decreased NREM sleep **(A,B)** and REM sleep **(D,E)** duration, increased latency to NREM sleep **(C)** and REM sleep **(F)**, and increased locomotor activity **(G)** Meloxicam had no significant effect on sleep duration, latency, or locomotor activity **(H–N)**. AT-403 increased NREM sleep duration **(O,P)** and decreased REM sleep duration **(R,S)**; latency was decreased to first NREM sleep bout **(Q)** and increased to first REM sleep bout **(T)** AT-403 acutely decreased locomotor activity during the light phase and increased activity during the dark phase **(U)** *p* < 0.05. Data expressed as the average ± SEM in error bars or shaded borders. Colored horizontal lines represent timepoints that are significant from vehicle at respective concentrations **(A,D,G,H,K,N,O,R,U)**; *, dose significant from vehicle (consistent with all other figures).

Prior to evaluating the interaction of pain and analgesics on sleep, we measured the effects of 3 different analgesics with distinct pharmacological mechanisms (MOP agonist, NSAID and NOP agonist) in pain-naïve states. Morphine (1.0–10.0 mg/kg) increased wake and decreased NREM and REM sleep duration both in the time course (primarily within the first 6 h post-dosing; Table 2 and Figures 3A,D) as well as in the summed 3-hour post-dosing period (Table 2 and Figures 3B,E). Morphine also dose-dependently increased NREM (1 and 3 mg/kg) and REM sleep latency (1, 3, 10 mg/kg; Table 2 and Figure 3F). For statistics, see Table 2. Consistent with increasing time awake, morphine also disrupted sleep continuity, noted by a significant decrease in the number of NREM sleep bouts (1.0, 3.0, 10.0 mg/kg; all *p* < 0.05) without affecting average NREM bout length, as well as decreased brief arousals (1.0, 3.0, 10.0 mg/kg; all *p* < 0.05). REM bout number (1.0, 3.0, 10.0 mg/kg; all *p* < 0.05 and average REM bout duration were also significantly decreased (3.0, 10.0 mg/kg; all *p* < 0.05; see Supplementary Figure S2 and Supplementary Table S2 for statistics). Lastly, morphine administration significantly reduced homecage locomotor activity, effects that persisted into the dark phase (10 mg/kg; see Table 5 for statistics, Figure 3G). As a control, the non-opioid receptor analgesic meloxicam was also evaluated on sleep. Consistent with clinical literature (48)^,^ meloxicam had no effect on wake (data not shown), NREM sleep, or REM sleep duration in the time course or 3 h post-dosing (Table 2, Figures 3H,I,K,L), did not impact NREM or REM sleep latency (Table 2, Figures 3J,M), and did not significantly impact any measures of sleep continuity (see Supplementary Figure S2, and Supplementary Table S2 for statistics). Meloxicam did not significantly affect homecage locomotor activity (Figure 3N).

**Table 2:** Effects of analgesics on sleep in male rats.

DV	Factor	DF	*F*	*p*	*	*Post hoc* results	Significant timepoints
Morphine (mg/kg) Mixed effects two-way ANOVA							
Wake (1 h bins)	Dose	5, 42	2.688	0.0340	*	0.1 mg/kg	None
	Time	12.73, 534.7	57.15	<0.0001	****	1 mg/kg	3, 4
	Int	115, 966	4.833	<0.0001	****	3 mg/kg	3, 4, 5, 6
						10 mg/kg	5, 6, 13
NREM (1 h bins)	Dose	5, 42	1.500	0.2105	ns	0.1 mg/kg	None
	Time	12.24, 514.2	55.42	<0.0001	****	1 mg/kg	3, 4
	Int	115, 966	4.464	<0.0001	****	3 mg/kg	3, 4, 5
						10 mg/kg	5, 6
REM (1 h bins)	Dose	5, 42	0.458	0.8053	ns	0.1 mg/kg	None
	Time	12.71, 533.8	34.88	<0.0001	****	1 mg/kg	4
	Int	115, 966	3.149	<0.0001	****	3 mg/kg	4, 5, 6, 15
						10 mg/kg	4, 5, 6, 7
Mixed effects one-way ANOVA							
Wake (3 h sum)	Dose	7	31.55	<0.0001	****	1, 3, 10 mg/kg	0.0002, 0.0002, 0.0080
NREM (3 h sum)	Dose	7	23.87	<0.0001	****	1, 3, 10 mg/kg	0.0005, 0.0003, 0.0237
REM (3 h sum)	Dose	7	32.27	<0.0001	****	1, 3, 10 mg/kg	0.0039, 0.0009, 0.0009
Latency to NREM	Dose	7	13.09	0.0008	***	1, 3, mg/kg	0.0005, 0.0013
Latency to REM	Dose	7	101.1	<0.0001	****	1, 3, 10 mg/kg	All <0.0001
Meloxicam (mg/kg) Mixed effects two-way ANOVA							
Wake (1 h bins)	Dose	3, 32	0.4608	0.7116	ns	0.5 mg/kg	None
	Time	6.212, 198.8	40.30	<0.0001	****	1 mg/kg	None
	Int	24, 256	0.7078	0.8424	ns	2 mg/kg	None
NREM (1 h bins)	Dose	3, 32	0.4666	0.7076	ns	0.5 mg/kg	None
	Time	6.209, 198.7	29.88	<0.0001	****	1 mg/kg	None
	Int	24, 256	0.7383	0.8098	ns	2 mg/kg	None
REM (1 h bins)	Dose	3, 32	1.108	0.3602	ns	0.5 mg/kg	8
	Time	5.928, 189.7	46.08	<0.0001	****	1 mg/kg	None
	Int	24, 256	1.588	0.0436	*	2 mg/kg	8
Mixed effects one-way ANOVA							
Wake (3 h sum)	Dose	8	0.6045	0.5761	ns	N/A	
NREM (3 h sum)	Dose	8	0.5660	0.6036	ns	N/A	
REM (3 h sum)	Dose	8	0.7159	0.5305	ns	N/A	
Latency to NREM	Dose	8	0.9773	0.4077	ns	N/A	
Latency to REM	Dose	8	0.2067	0.8125	ns	N/A	
AT-403 (mg/kg) Mixed effects two-way ANOVA							
Wake (1 h bins)	Dose	4	4.896	0.0015	**	0.01 mg/kg	3, 4, 8
	Time	10.72	105.1	<0.0001	****	0.03 mg/kg	3, 16
	Int	92	2.828	<0.0001	****	0.10 mg/kg	3, 4
						0.30 mg/kg	3–6, 21
NREM (1 h bins)	Dose	3.472, 48.61	34.65	<0.0001	****	0.01 mg/kg	3, 4, 7
	Time	6.568, 91.96	115.2	<0.0001	****	0.03 mg/kg	3–5, 7
	Int	10.91, 152.7	5.828	<0.0001	****	0.10 mg/kg	3–10, 16
						0.30 mg/kg	3–11, 13, 21
REM (1 h bins)	Dose	4, 70	28.68	<0.0001	****	0.01 mg/kg	4
	Time	12.36, 865.4	28.44	<0.0001	****	0.03 mg/kg	3–5
	Int	92, 1610	6.155	<0.0001	****	0.10 mg/kg	3–7, 9, 24
						0.30 mg/kg	4–11, 21, 23
Mixed effects one-way ANOVA							
Wake (3 h sum)	Dose	2.434, 34.08	43.74	<0.0001	ns	0.03, 0.10, 0.30	All <0.0001
NREM (3 h sum)	Dose	2.636, 36.90	75.38	<0.0001	ns	0.01, 0.03, 0.10, 0.30	All <0.0001
REM (3 h sum)	Dose	2.330, 32.63	49.78	<0.0001	****	0.01, 0.03, 0.10,0.30	0.0418, <0.0001, <0.0001, <0.0001
Latency to NREM	Dose	4, 14	36.87	<0.0001	****	0.03, 0.10, 0.30	All <0.0001
Latency to REM	Dose	4, 14	175.8	<0.0001	****	0.03, 0.10, 0.30	0.0152, <0.0001, <0.0001

ns, not significant; h, hour; Int, interaction.

* *p* < 0.05.

** *p* < 0.01.

*** *p* < 0.001.

**** *p* < 0.0001.

**Table 5:** Effects of analgesics on quantitative EEG in male rats.

DV	Factor	DF	*F*	*p*	*	*Post hoc* results	Significant timepoints
Mixed effects two-way ANOVA							
Morphine (mg/kg)							
Delta (Wake)	Dose	1.718,12.02	9.579	0.0042	**	10 mg/kg	6, 8
	Time	2.962,20.73	6.903	0.0022	**		
	Int	4.675,32.72	1.141	0.3575	ns		
Beta (Wake)	Dose	1.210,8.469	9.575	0.0114	*	1 mg/kg	4
	Time	2.776,19.43	5.852	0.0059	**	10 mg/kg	5, 8
	Int	3.523,24.66	2.141	0.1125	ns		
High Gamma (Wake)	Dose	1.967,13.77	35.04	<0.0001	****	1 mg/kg	4, 5
	Time	2.971,20.80	3.128	0.0480	*	3 mg/kg	4, 5
	Int	4.671,32.70	11.82	<0.0001	****	10 mg/kg	5, 6
Delta (NREM)	Dose	5, 35	7.8	<0.0001	****	1 mg/kg	4, 5
	Time	7, 49	5.495	0.0001	***	3 mg/kg	4, 5
	Int	35, 186	4.212	<0.0001	****	10 mg/kg	5, 6
Sigma (NREM)	Dose	5, 35	6.303	0.0003	***	3 mg/kg	8
	Time	5, 35	7.495	<0.0001	****	10 mg/kg	8
	Int	25, 117	4.626	<0.0001	****		
Locomotor activity	Dose	2.066, 14.46	1.909	0.1830	ns	0.1 mg/kg	1
	Time	3.821, 26.75	14.50	<0.0001	****	1 mg/kg	4
	Int	5.082, 35.57	2.774	0.0317	*	3 mg/kg	4–5
						10 mg/kg	5–6, 13–15
Meloxicam (mg/kg)							
Delta (Wake)	Dose	1.943,15.55	0.7104	0.5029	ns	N/A	
	Time	1.606,12.85	0.7720	0.4555	ns		
	Int	3.521,28.17	1.110	0.3672	ns		
Beta (Wake)	Dose	1.397,11.18	0.6261	0.4973	ns	None	
	Time	2.038,16.30	5.095	0.0187	*		
	Int	4.083,32.66	0.5890	0.6762	ns		
High Gamma (Wake)	Dose	2.058,16.47	0.1390	0.8766	ns	None	
	Time	3.001,24.01	11.20	<0.0001	****		
	Int	4.049,32.39	1.151	0.3508	ns		
Delta (NREM)	Dose	1.771,14.17	0.9049	0.4152	ns	None	
	Time	2.310,18.48	11.87	0.0003	***		
	Int	4.254,34.03	1.054	0.3966	ns		
Sigma (NREM)	Dose	1.834,14.67	0.8752	0.4288	ns	2 mg/kg	5, 6
	Time	1.815,14.52	8.465	0.0044	**		
	Int	4.939,39.51	0.7086	0.6189	ns		
Locomotor activity	Dose	2.631, 21.05	0.1423	0.9151	ns	None	
	Time	4.504, 36.03	20.03	<0.0001	****		
	Int	4.913, 39.30	0.8929	0.4938	ns		
AT-403 (mg/kg)							
Delta (Wake)	Dose	2.683, 37.56	13.80	<0.0001	****	0.03 mg/kg	14
	Time	2.868, 40.15	13.36	<0.0001	****	0.10 mg/kg	7–12, 24
	Int	5.467, 74.11	4.894	0.0004	***	0.30 mg/kg	6–13
Beta (Wake)	Dose	2.749, 38.49	11.57	<0.0001	****	0.10 mg/kg	5–9
	Time	2.389, 33.45	9.363	0.0003	***	0.30 mg/kg	6–11
	Int	5.827, 78.98	10.15	<0.0001	****		
High Gamma (Wake)	Dose	3.294, 46.11	7.383	0.0003	***	0.03 mg/kg	5, 16
	Time	6.054, 84.76	60.24	<0.0001	****	0.10 mg/kg	5–8, 13, 24
	Int	9.577, 129.8	2.674	0.0059	**	0.30 mg/kg	3, 6–14, 23
Delta (NREM)	Dose	1.964, 27.49	34.49	<0.0001	****	0.03 mg/kg	10–13
	Time	4.850, 67.90	27.98	<0.0001	****	0.10 mg/kg	3–13, 23
	Int	7.089, 89.69	5.464	<0.0001	****	0.30 mg/kg	3–17, 21–23
Sigma (NREM)	Dose	2.349, 32.88	21.50	<0.0001	****	0.03 mg/kg	4, 5,
	Time	4.920, 68.87	32.13	<0.0001	****	0.10 mg/kg	4–12, 23
	Int	5.742, 72.65	4.473	0.0008	***	0.30 mg/kg	1–17, 21–23
Locomotor activity	Dose	2.654, 37.15	2.469	0.0838	ns	0.01 mg/kg	
	Time	5.346, 74.85	47.27	<0.0001	****	0.03 mg/kg	3, 16
	Int	9.800, 137.2	2.082	0.0309	*	0.10 mg/kg	3, 18
						0.30 mg/kg	3–5, 20–21

ns, not significant; Int, interaction.

* *p* < 0.05.

** *p* < 0.01.

*** *p* < 0.001.

**** *p* < 0.0001.

Interestingly, AT-403 significantly increased NREM sleep and decreased wake at all doses, both over time (Table 2, Figure 3O) (effects lasting approximately 6 h post-dosing) and in the summed 3-hour bin post-dosing (Table 2, Figure 3P). However, REM sleep was consequently decreased over time (Table 2, Figure 3R), and in the summed 3-hour post-dosing bin (Table 2 and Figure 3S) at all doses. Corresponding dose-dependent decreases in NREM sleep latency (0.03–0.30 mg/kg) and increases in REM sleep latency (0.03–0.30 mg/kg) were also observed (Table 2 and Figures 3Q,T). AT-403 significantly decreased homecage locomotor activity acutely, with significant increases in activity noted during the dark phase (Table 5 and Figure 3U). AT-403 significantly decreased brief arousals (0.1, 0.3 mg/kg) and average NREM sleep bout number (0.01–0.3 mg/kg) but significantly increased average NREM sleep bout duration (0.03–0.3 mg/kg) while both REM sleep bout frequency (0.03–0.3 mg/kg), and average REM sleep bout duration (0.01–0.3 mg/kg) were significantly decreased (all *p* < 0.05 compared to respective vehicle; see Supplementary Figure S2, and Supplementary Table S2 for statistics).

### Effects of analgesics + lactic acid on pain-associated behaviors

**Figure 4:**
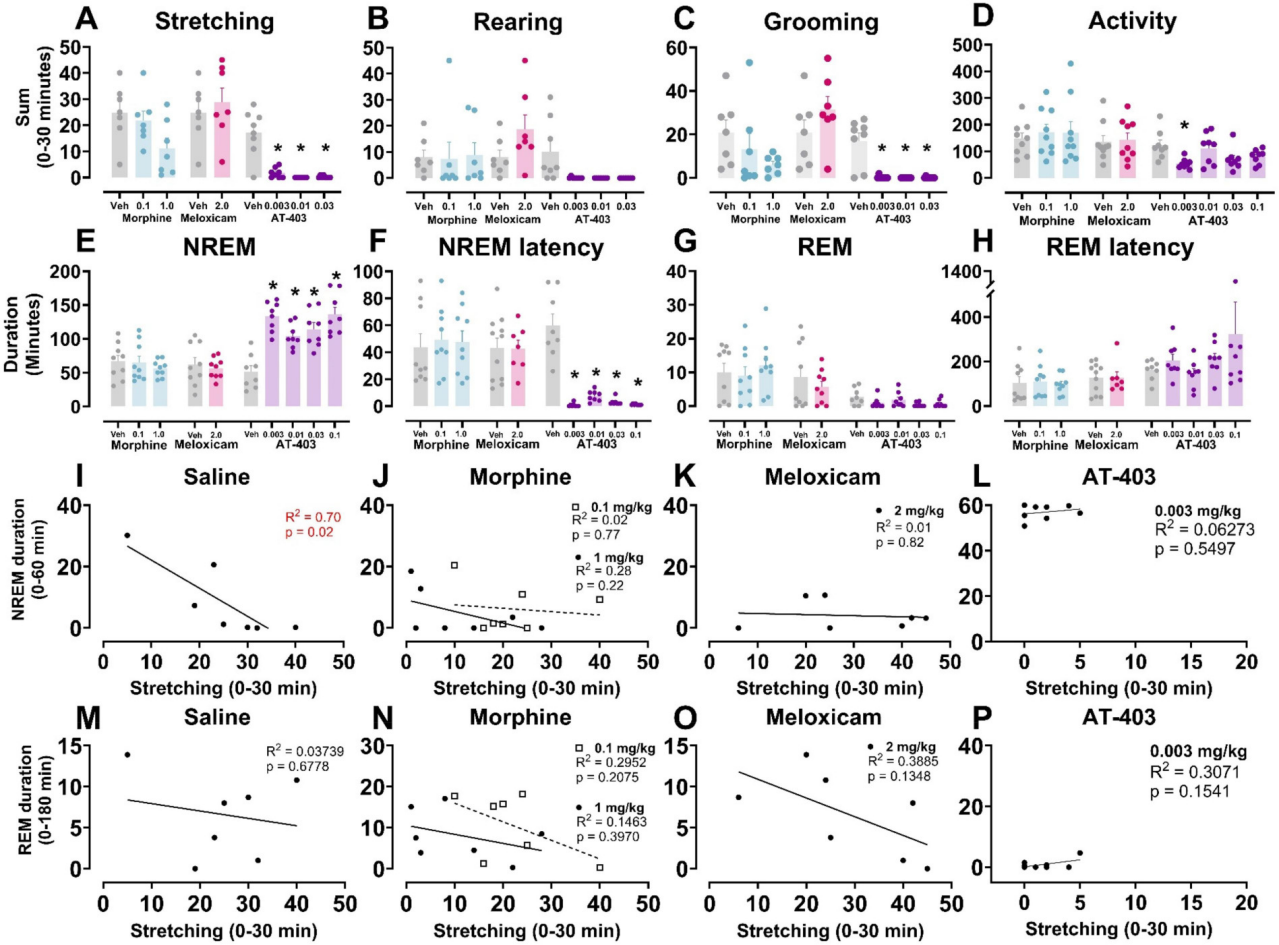
AT-403 prevented lactic acid-induced sleep disruption but not morphine or meloxicam. In panels **(A–H)** saline, morphine, meloxicam, or AT-403 was administered 1 h prior to 5.6% lactic acid and sleep or behavior was recorded. AT-403, but not morphine or meloxicam, decreased pain-related behaviors stretching **(A)** and grooming **(C)** 30 min post-administration but not rearing **(B)** or spontaneous homecage locomotor activity sum during EEG recording 30 min post-administration **(D)** AT-403, but not morphine or meloxicam, increased NREM sleep **(E)** and decreased REM sleep **(G)** duration 3 h post administration, and decreased NREM sleep latency **(F)** but not REM sleep latency **(H)** Significant correlations between NREM sleep (1 h post-lactic acid administration) or REM sleep duration (3 h post administration) and stretching (0–30 min post lactic acid administration) were present following saline **(I,M)** but not morphine **(J,N)**, meloxicam **(K,O)**, or AT-403 administration **(L,P)**. Data expressed as the average ± SEM in error bars. * Dose significant from vehicle (*p* < 0.05). *R*^2^ and *p* values are shown in red (significant *p* < 0.05) or black (not significant).

We then determined if a pre-treatment of an analgesic (morphine, meloxicam, or AT-403) would alter pain-associated behaviors following 5.6% lactic acid administration. A main effect of AT-403 was found on stretching, rearing, and grooming while meloxicam and morphine had no effect. *Post hoc* analyses revealed that AT-403 decreased stretching and grooming behaviors at 0.003, 0.01, and 0.03 mg/kg (Table 3, Figures 4A–C). AT-403, but not morphine or meloxicam, decreased spontaneous locomotor activity as recorded from EEG transmitters (Table 3, Figure 4D).

**Table 3:** Effects of analgesics + 5.6% lactic acid on sleep in male rats.

DV	Factor	DF	*F*	*p*	*	*Post hoc* results	Significant timepoints
Morphine + 5.6% lactic acid							
Mixed effects two-way ANOVA							
Wake (1 h bins)	Dose	1.805, 14.44	0.03829	0.9513	ns	0.5 mg/kg	None
	Time	3.471, 27.77	0.03829	<0.0001	****	1 mg/kg	None
	Int	4.891, 39.13	1.302	0.2835	ns		
NREM (1 h bins)	Dose	1.734, 13.87	0.1938	0.7960	ns	0.5 mg/kg	None
	Time	3.273, 26.18	36.63	<0.0001	****	1 mg/kg	None
	Int	4.951, 39.60	1.099	0.3761	ns		
REM (1 h bins)	Dose	1.949, 15.59	5.433	0.0167	*	0.5 mg/kg	None
	Time	2.965, 23.72	22.45	<0.0001	****	1 mg/kg	5, 7, 9
	Int	5.210, 41.68	2.308	0.0591	ns		
Mixed effects one-way ANOVA							
Wake (3 h sum)	Dose	1.721, 13.77	0.3499	0.6798	ns	N/A	
NREM (3 h sum)	Dose	1.782, 14.25	0.8224	0.4463	ns	N/A	
REM (3 h sum)	Dose	1.614, 12.91	0.6388	0.5115	ns	N/A	
NREM latency	Dose	1.962, 15.69	0.1363	0.8699	ns	N/A	
REM latency	Dose	1.816, 14.53	0.9080	0.8008	ns	N/A	
Stretching	Dose	1.501, 13.51	3.361	0.0759	ns	N/A	
Rearing	Dose	1.185, 7.109	0.2329	0.9149	ns	N/A	
Grooming	Dose	1.510, 13.59	1.854	0.1970	ns	N/A	
Activity	Dose	1.323, 10.58	0.1983	0.7325	ns	N/A	
Meloxicam + 5.6% lactic acid							
Mixed effects two-way ANOVA							
Wake (1 h bins)	Dose	3, 24	2.739	0.0656	ns	none	
	Time	8, 64	40.50	<0.0001	****		
	Int	24, 156	1.176	0.2718	ns		
NREM (1 h bins)	Dose	1, 8	0.4780	0.5089	ns	none	
	Time	8, 64	23.04	<0.0001	****		
	Int	8, 64	0.9093	0.5146	ns		
REM (1 h bins)	Dose	1, 8	0.2191	0.6522	ns	none	
	Time	<0.0001	16.91	<0.0001	****		
	Int	8, 64	0.5664	0.8014	ns		
Two-tailed paired *t*-test							
	Factor	DF	*t*	*p*	*		
Wake (3 h sum)	Dose	8	0.6475	0.5354	ns		
NREM (3 h sum)	Dose	8	0.5268	0.6126	ns		
REM (3 h sum)	Dose	8	1.038	0.3296	ns		
NREM latency	Dose	6	0.3251	0.7561	ns		
REM latency	Dose	6	0.3556	0.9728	ns		
Stretching	Dose	6	0.5829	0.4809	ns		
Rearing	Dose	6	1.423	0.2046	ns		
Grooming	Dose	6	1.445	0.1986	ns		
Activity	Dose	8	0.2019	0.8451	ns		
AT-403 + 5.6% lactic acid AT-403							
Mixed effects two-way ANOVA							
Wake (1 h bins)	Dose	1.759, 12.31	13.63	0.0010	***	0.003 mg/kg	1–5
	Time	2.697, 18.88	5.368	0.0091	**	0.01 mg/kg	3–4
	Int	4.629, 32.40	8.426	<0.0001	****	0.03 mg/kg	2–4
						0.10 mg/kg	2–4
NREM (1 h bins)	Dose	1.783, 12.48	18.85	0.0002	***	0.003 mg/kg	2–4
	Time	2.838, 19.86	48.93	<0.0001	****	0.01 mg/kg	2–4
	Int	5.117, 35.82	6.878	0.0001	***	0.03 mg/kg	1–4
						0.10 mg/kg	2–4
REM (1 h bins)	Dose	2.766, 19.36	3.850	0.0282	*	0.003 mg/kg	2, 8
	Time	2.212, 15.49	23.13	<0.0001	****	0.01 mg/kg	2
	Int	5.061, 35.42	2.525	0.0463	*	0.03 mg/kg	3
						0.10 mg/kg	1–2, 8
Mixed effects one-way ANOVA							
Wake (3 h sum)	Dose	1.930, 13.51	28.98	<0.0001	****	0.003, 0.01, 0.03, 0.1	<0.0001, <0.0001, 0.0015, 0.0001
NREM (3 h sum)	Dose	1.885, 13.19	30.85	<0.0001	****	0.003, 0.01, 0.03, 0.1	<0.0001, <0.0001, 0.0012, <0.0001
REM (3 h sum)	Dose	2.176, 15.23	2.491	0.1126	ns	N/A	
NREM latency	Dose	1.079, 7.551	43.21	0.0002	***	0.003, 0.01, 0.03, 0.1	0.0007, 0.0014, 0.0011, 0.0008
REM latency	Dose	1.133, 9.913	1.063	0.3377	ns	N/A	
Stretching	Dose	1.058, 7.405	30.06	0.0007	***	0.003, 0.01, 0.03	0.0020, 0.0022, 0.0023
Rearing	Dose	1.002, 9.347	5.789	0.0385	*	None	
Grooming	Dose	1.009, 9.418	20.33	0.0013	**	0.003, 0.01, 0.03	0.0067, 0.0066, 0.0077
Activity	Dose	2.704, 18.93	7.172	0.0026	**	0.003	0.0209

ns, not significant. Stretching, rearing, and grooming behaviors are summed during the first 30 min after lactic acid administration; h, hour; Int, interaction.

* *p* < 0.05.

** *p* < 0.01.

*** *p* < 0.001.

**** *p* < 0.0001.

### Effects of analgesics + lactic acid on sleep

**Figure 5:**
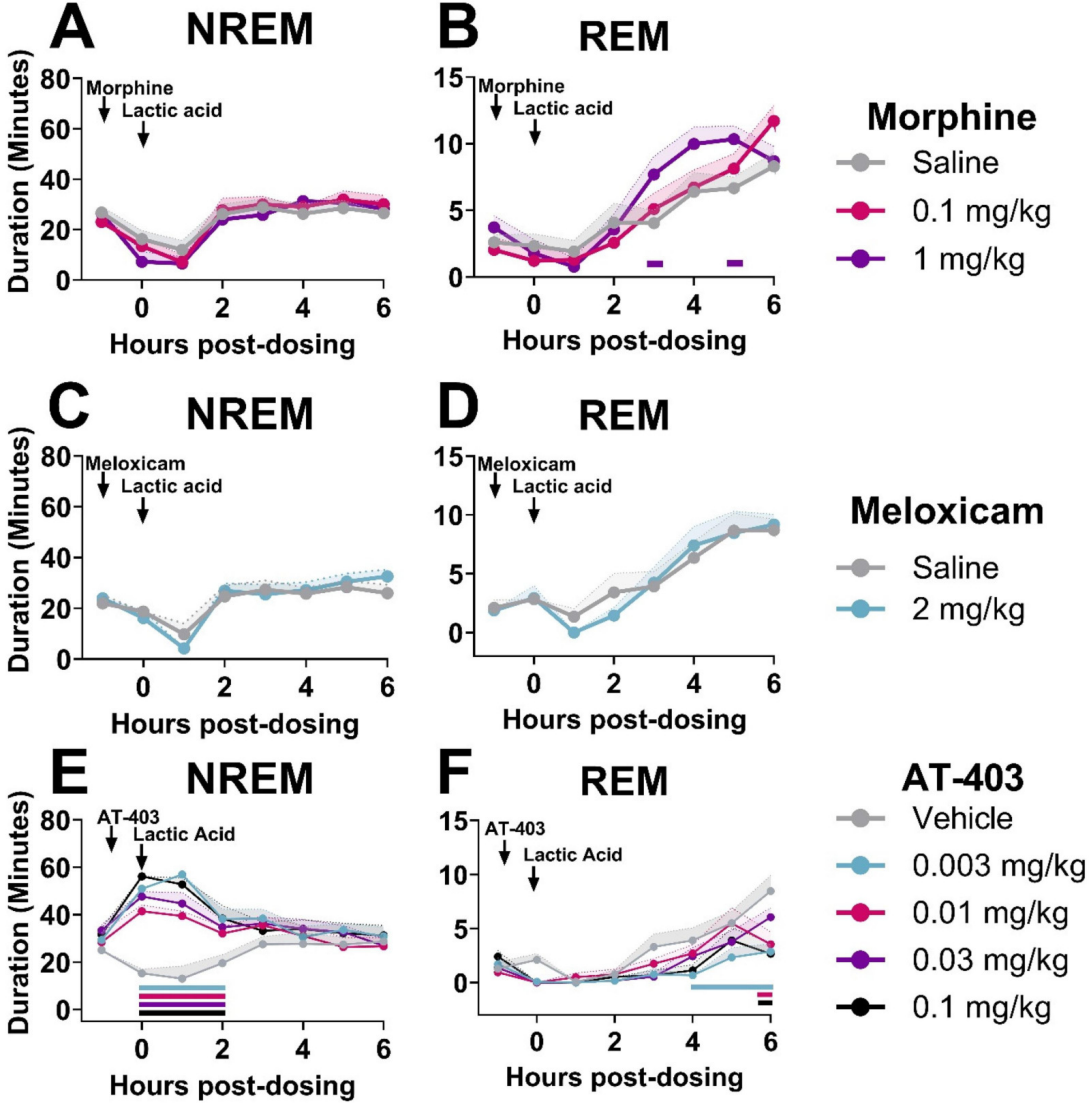
AT-403, but not morphine or meloxicam, prevented lactic acid-induced sleep disturbances. NREM and REM sleep duration in 1-hour bins over the 6 h post-dosing period. Arrows indicate when each compound and lactic acid was administered, with analgesics administered 1 h prior to 5.6% lactic acid at timepoint 0. Morphine did not affect NREM sleep **(A)**, but decreased REM sleep **(B)** sleep following lactic acid administration. Meloxicam did not influence NREM sleep **(C)** or REM **(D)** sleep at any dose. AT-403 promoted NREM **(E)**, but decreased REM sleep **(F)** following lactic acid administration. *p* < 0.05. Data expressed as the average ± SEM in shaded borders; horizontal lines corresponding to respective dose color represent a significant from vehicle/saline + 5.6% lactic acid at associated timepoints.

In order to determine if analgesics with distinct mechanisms alleviate or further disrupt lactic acid-induced sleep disruption, EEG was evaluated following administration of morphine, meloxicam, or AT-403 1 h prior to 5.6% lactic acid. There was no acute effect of morphine or meloxicam administration on lactic acid-induced NREM sleep disruptions (Table 3 and Figures 4E, 5A,C), but 1 mg/kg morphine increased REM sleep at specific timepoints during the 6-hour post-dosing period (Table 3 and Figure 5B). Conversely, AT-403 significantly increased NREM sleep and decreased wake and REM sleep over time even with lactic acid present, and *post hoc* analyses revealed increases in NREM sleep, and decreases in wake (data not shown) and REM sleep across all doses for approximately 2 (NREM sleep) to 6 (REM sleep) hours (Table 3 and Figures 5E,F). Additionally, paired *t*-tests or one-way ANOVAs were conducted to assess the effects of morphine, meloxicam, and AT-403 on NREM and REM sleep duration and sleep continuity for the 3-hour duration post lactic acid administration. AT-403, but not morphine or meloxicam, produced a main effect on wake (data not shown), and NREM sleep at all doses, and a decrease in REM sleep occurred during the 3-hour post-dosing period (Table 3 and Figures 4E,G). Paired *t*-tests or one-way ANOVAs on NREM & REM sleep latency revealed no significant differences between the vehicle and meloxicam or morphine groups, but NREM sleep latency was reduced following AT-403 at all doses (Table 3 and Figures 4F,H). Regarding sleep continuity, AT-403 administration prior to 5.6% lactic acid produced a significant main effect of dose on NREM sleep bout frequency although no dose reached statistical significance compared to lactic acid administration alone (all *p* > 0.05); no other measures were affected. Similarly, neither morphine nor meloxicam, in combination with 5.6% lactic acid affected sleep continuity measures (see Supplementary Figure S1 and Supplementary Table S1 for statistics).

### Correlations between lactic acid-induced acute abdominal pain and effects of lactic acid on sleep

To determine whether pain and sleep measures correlated with one another, and whether analgesics would influence the relationship between pain and sleep, within-subject linear regressions were conducted between stretching (summed 0–30 min post lactic acid administration) and NREM sleep (summed 0–60 min post-lactic acid administration) or REM sleep (summed 0–180 min post-lactic acid administration) duration. We found that stretching correlated with NREM sleep when pre-treated with saline 1 h prior to lactic acid administration but this correlation was lost when pre-treated with morphine, meloxicam, or AT-403 (Table 4 and Figures 4I–L). Interestingly, there were no significant correlations between REM sleep and stretching (Table 4 and Figures 4M–P).

**Table 4:** Relationship between stretching and NREM or REM duration following administration of analgesics + lactic acid.

DV	Factor	DF	*F*	*R* ^2^	*p*	*
Simple linear regression						
Stretching × NREM	Saline (i.p.) + 5.6% LA	1, 5	11.79	0.7022	0.0186	*
Stretching × REM	Saline (i.p.) + 5.6% LA	1, 5	0.1942	0.03739	0.6778	ns
Stretching × NREM	2 mg/kg Meloxicam + 5.6% LA	1, 5	0.05723	0.01132	0.8204	ns
Stretching × REM	2 mg/kg Meloxicam + 5.6% LA	1, 5	3.176	0.3885	0.1348	ns
Stretching × NREM	0.5 mg/kg morphine + 5.6% LA	1, 5	0.09159	0.01799	0.7744	ns
Stretching × NREM	1 mg/kg morphine + 5.6% LA	1, 5	1.919	0.2774	0.2246	ns
Stretching × REM	0.5 mg/kg morphine + 5.6% LA	1, 5	2.094	0.2952	0.2075	ns
Stretching × REM	1 mg/kg morphine + 5.6% LA	1, 5	0.8571	0.1463	0.3970	ns
Stretching × NREM	0.003 mg/kg AT-403 + 5.6% LA	1, 6	0.4015	0.06273	0.5497	ns
Stretching × REM	0.003 mg/kg AT-403 + 5.6% LA	1, 6	2.659	0.3071	0.1541	ns

ns, not significant. Stretching, rearing, and grooming behaviors are summed during the first 30 min after lactic acid administration. NREM 0–60 min, REM 0–120 min.

* *p* < 0.05.

### The effects of lactic acid and analgesics alone and in combination on quantitative EEG

**Figure 6:**
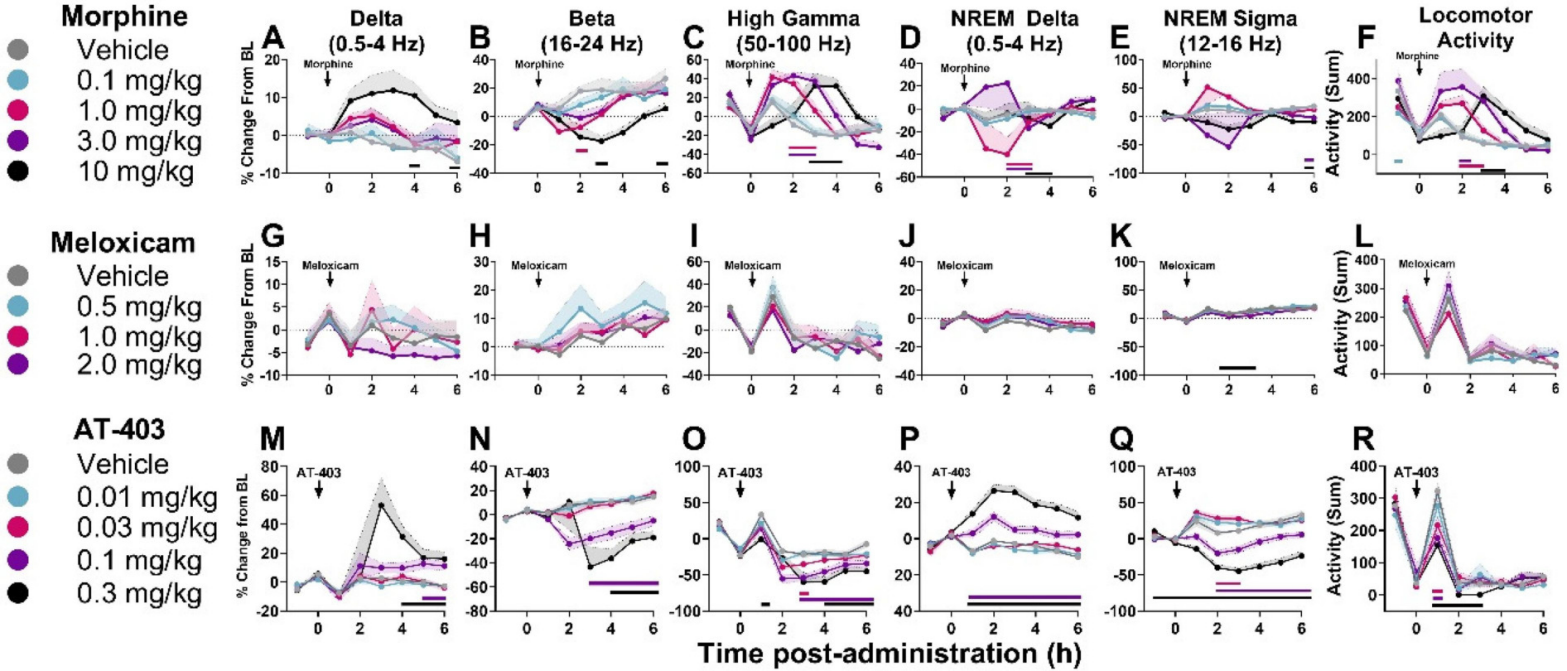
Effects of morphine, meloxicam, and AT-403 on quantitative EEG and locomotor activity. Data expressed as percent change from within-subjects same-day baseline for 6 h post-dosing. Morphine increased delta, decreased beta, increased high gamma power, and increased locomotor activity **(A–F)**. Meloxicam decreased NREM sleep sigma power but otherwise had no effect on quantitative EEG activity **(G–L)**. AT-403 increased delta, decreased beta, decreased high gamma, increased NREM sleep delta, increased NREM sleep sigma, and decreased locomotor activity **(M–R)**. Locomotor activity data are replotted from Figure 3 to highlight the acute effects of morphine **(F)** and AT-403 **(R)** during the first 6 h post-administration. Data expressed as the average ± SEM in shaded borders; colored horizontal lines signify dose significant from vehicle at those timepoints.

When administered alone, lactic acid decreased alpha power during wake 2 h post-dosing in males, but there were no significant effects of lactic acid on brain function in females (Supplementary Tables S3, S4 and Supplementary Figure S3). Morphine, meloxicam, and AT-403 produced divergent effects on brain function. At lower doses, morphine increased high gamma and decreased beta power during wake (Table 5 and Figures 6B,C). At the highest dose, morphine increased delta power during wake, and high gamma power was not elevated during that time period (Table 5 and Figures 6A,C), suggesting a sedative profile. During NREM sleep, morphine dose-dependently influenced NREM sleep delta and sigma power (Table 5 and Figures 6D,E). Meloxicam decreased NREM sleep sigma power for approximately 2 h (Table 5 and Figure 6K). AT-403 increased waking and NREM sleep delta power and decreased waking beta, high gamma, and NREM sigma power (Table 5 and Figures 6M–R) during the first 6 h post-administration; qEEG changes during NREM sleep persisted for 6–8 h post-administration and waking EEG took place 4–6 h post-administration. See Supplementary Tables S5–S7 and Supplementary Figures S4, S5 for effects on additional waveforms.

Lastly, quantitative EEG activity following pre-treatment with analgesics morphine, meloxicam, and AT-403, followed by 5.6% lactic acid, was also evaluated. Pre-treatment with morphine, meloxicam, or AT-403 prior to lactic acid-administration did not affect quantitative EEG waveforms affected by lactic acid alone (Supplementary Figure S3 and Supplementary Tables S9–S11). However, pre-treatment of AT-403 prior to lactic acid-administration produced subtle to moderate increases in theta and alpha, and decreases in sigma, low gamma, high gamma, and total gamma power during waking periods. During NREM sleep, AT-403 decreased sigma power compared to lactic acid alone (0.003 mg/kg, 0.1 mg/kg *p* < 0.05; Supplementary Figure S5 and Supplementary Table S11).

### Effects of AT-403 on locomotor activity

**Figure 7:**
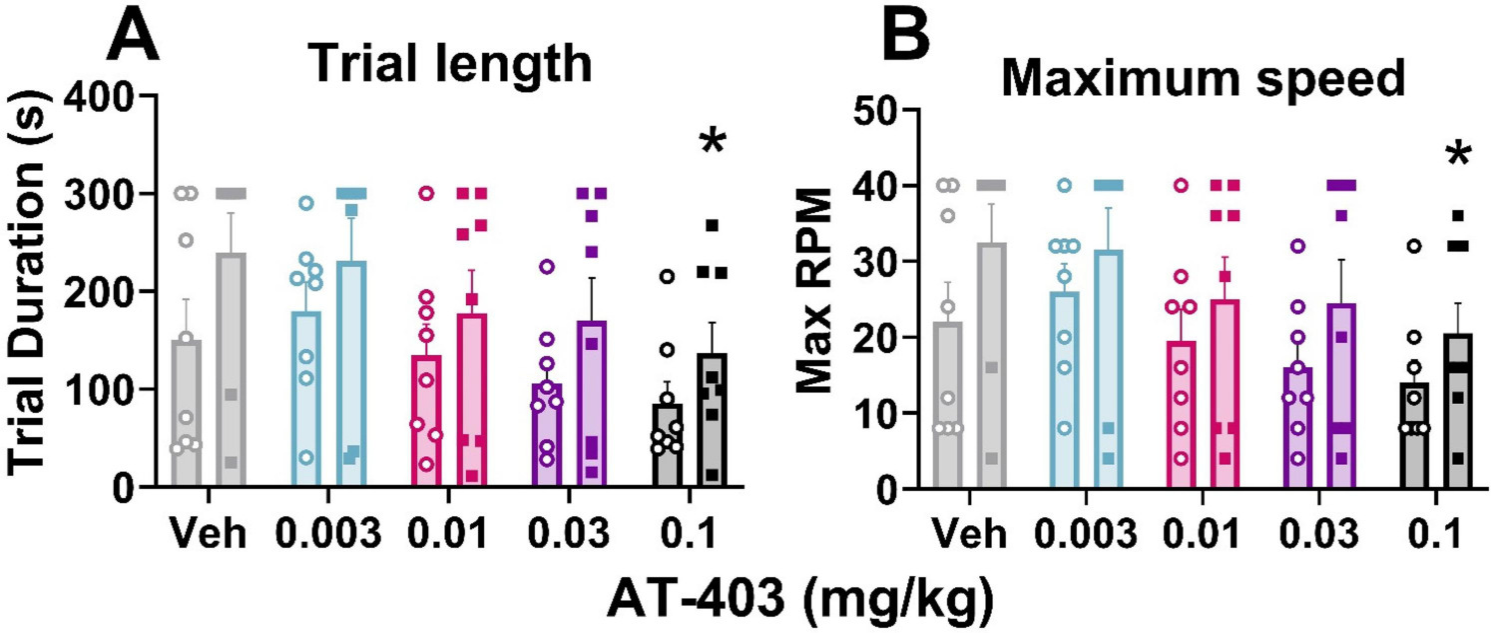
Effects of AT-403 on locomotor function. The highest dose of AT-403 decreased trial duration **(A)** and maximum speed/rotations per minute (RPM) reached **(B)** on the Rotarod task in females (squares) but not males (open circles). No sex differences were detected. Data expressed as the average ± SEM in error bars. *p < 0.05, significant from vehicle.

To determine whether AT-403 produced locomotor deficits, rats were administered AT-403 or vehicle 1 h prior to being placed on the accelerating Rotarod device until the rat fell off or 5 min was reached. A main effect of dose was found for both trial duration (*F* = 6.790, *p* = 0.0002) and maximum RPM (*F* = 5.990, *p* = 0.0004). Dunnett's *post hoc* tests were used to determine significant doses from vehicle and between males and females, which revealed that 0.1 mg/kg AT-403 decreased trial duration in females (*p* = 0.0046) but not males (*p* > .05) (Figure 7A). Similarly, 0.1 mg/kg AT-403 decreased maximum RPM in females (*p* = 0.0122) but not males (*p* > .05) (Figure 7B).

## Discussion

Sleep plays a direct role in pain management and recovery, and pain-induced sleep disturbances are not adequately targeted by current analgesics; in fact, current medications often disrupt sleep (9, 91, 92). In order to target this unmet need, we investigated a NOP agonist, a novel and promising approach that has shown antinociceptive effects and promotes sleep in pain-naïve subjects. The present study examined the effects of lactic acid, an acute noxious stimulus, on pain-associated behaviors and sleep both alone and in combination with morphine (MOP full agonist), meloxicam (NSAID) or AT-403 (NOP agonist). As expected, lactic acid increased pain-related behaviors (79, 80, 82) (Figures 1A–C) and, importantly, transiently decreased NREM/REM sleep duration (Figures 2A–D) and increased NREM and REM sleep latencies (Figures 2E,F). While morphine (Figures 3A,B) and meloxicam (Figures 3H,I) disrupted NREM sleep or had no effect in pain-naïve states, respectively, AT-403 promoted NREM sleep (Figures 3O,P). Moreover, only AT-403 effectively overcame lactic acid-induced reductions in NREM sleep (Figures 4, 5). Together, these data demonstrate that current analgesics do not sufficiently ameliorate acute pain-induced sleep disturbances and support the development of NOP agonists for the treatment of acute pain-associated sleep disturbances.

Consistent with other acute pain models (72, 74, 75, 77), we found that lactic acid acutely disrupted sleep (Figure 2). Interestingly, although females were more sensitive to the behavioral effects of lactic acid, males were more sensitive to the disruptive effects on NREM sleep. In males, decreases in NREM and REM sleep were present for approximately 2 and 3 h, respectively (Figures 2C–F). In females, no changes in NREM sleep were detected, but decreases in REM sleep persisted for 2–4 h (Figure 2). These results are consistent with clinical literature reporting distinct sex differences in pain sensitivity, pain disorder prevalence, and pain symptom clusters, including heightened pain sensitivity in females whereas males are more susceptible to pain-induced sleep disturbance (93–96). Due to more prominent sleep-disrupting effects in males than in females, subsequent lactic acid studies only included males; future studies should extend these studies to female subjects. In males, NREM sleep duration following 5.6% lactic acid administration was inversely correlated with stretching (Figure 4I). While clinical literature shows acute and chronic pain-related changes in qEEG (97–99) and that qEEG could be used as a translational biomarker for pain, we did not identify any lactic acid-induced changes in males or females during waking durations (Supplementary Figure S3), although transient reductions in delta power during NREM sleep suggest reduced sleep quality (Supplementary Figure S5). Overall, these findings demonstrate that lactic acid produced sleep disruptions consistent with other preclinical acute pain models (72, 74, 75, 77), yet may not be noxious enough to recapitulate qEEG changes during waking states associated with more severe pain conditions in humans.

Despite being highly effective analgesics, patients report that MOP agonists and NSAIDs do not improve and may even exacerbate pain-associated sleep disturbances (9, 91, 92). These findings have not been well-characterized in preclinical models. As shown in our study, morphine disrupted sleep by increasing NREM & REM sleep latency (Figures 3C,F) and decreasing NREM and REM sleep duration (Figures 3A,B,D,E) for approximately 3–4 h in pain-naïve rats as well as bout number (Supplementary Figure S2), consistent with previous studies (38, 40, 100, 101). Importantly however, our data show that the duration of these sleep disruptions in the pain-naïve state overlaps with and persists beyond morphine's anti-nociceptive activity (2–3 h) (102), suggesting that at the dose range tested the sleep-disrupting effects may outweigh the potential analgesic effects. The analgesic effects of MOP agonists, including morphine, are mediated through inhibition and activation of the ascending and descending pain pathways, respectively, to prevent pain signals in the dorsal root ganglion (DRG) of the spinal cord from reaching the brain (103, 104). In contrast, the sleep-disrupting effects of morphine are driven through activation of wake-promoting systems in the hypothalamus, orexin system, and locus coeruleus, and through inhibition of sleep-promoting circuitry in the hypothalamus, ventrolateral preoptic nucleus, and median preoptic nucleus (34, 105). Although morphine failed to alleviate lactic-acid induced sleep disruptions in the current studies, it did not worsen sleep either (Figures 4E,G, 5A,B). In contrast, meloxicam did not alter lactic-acid induced sleep disruptions nor augment sleep or spectral EEG (Figures 4E,G, 5C,D and Supplementary Figures S3–S5), consistent with a peripheral analgesic mechanism of action via COX receptor inhibition (44). However, low NREM/REM sleep durations based on handling/activity in proximity to lactic acid administration may have precluded our ability to detect further sleep disruptions and need to be examined in more long-lasting models of moderate or chronic pain states. Regardless, these data align with clinical literature in which patients prescribed MOP agonists and NSAIDs continue to report sleep disruption despite effective analgesia (9, 91, 92).

In contrast, the NOP agonist AT-403 demonstrated a unique profile for the treatment of pain-induced sleep disturbances. Consistent with prior literature examining other NOP agonists (69, 70), we found that AT-403 significantly increased NREM sleep duration and average bout length while decreasing REM sleep duration and bout number/average length at higher doses (Figures 3O–T and Supplementary Figure S2). While not directly examined in the present studies, NOP agonists have been shown to decrease activity in the hypothalamus, suprachiasmatic nucleus (SCN), and hypocretin/orexin system (70, 106–109): all of which are known to promote wakefulness (106, 110–113) and likely drive their sleep-promoting effects (34, 105). Unlike morphine and meloxicam, AT-403 extinguished all tested behaviors after lactic acid administration (Figures 4A,C) and, similar to a pain-naïve state, promoted NREM sleep compared to lactic-acid administration alone (Figures 4E, 5G,E,F). While NOP agonists have been effective in mitigating both acute and chronic pain in several animal models (61, 64, 114), and AT-403 increased NREM sleep duration in the presence of lactic acid, sleep continuity measures were not significantly improved. Further studies are needed to understand the dual effects of NOP agonists like AT-403 in more severe and longer-lasting models of pain-induced sleep disruptions.

While NREM sleep following lactic acid administration inversely correlated with stretching (Figure 4I), this relationship was lost following pre-treatment of morphine, meloxicam, or AT-403 (Figures 4J–L). We hypothesized that AT-403 would have analgesic and sleep-promoting effects resulting in a stronger correlation and steeper slope between pain and sleep measures (e.g., stretching would be reduced and NREM sleep would be increased), and meloxicam might have maintained a similar relationship via analgesic efficacy alone resulting in improved sleep. In contrast, we expected that morphine would have analgesic yet sleep disrupting effects (resulting in a leftward and downward shift in the linear regression). There are several plausible explanations for our outcomes. Lactic acid administration is associated with pain-induced (stretching) and pain-suppressed behaviors (rearing, grooming), yet at higher doses all behavior is suppressed. Our selection of 5.6% lactic acid is on the descending limb of the dose-response curve regarding pain-induced behavior (115), and in fact was not different from vehicle-associated stretching in female rats. Thus, it is not surprising that morphine and meloxicam did not significantly alter stretching. In fact, analgesic effects of any drug/compound could manifest as an increase in stretching behavior in combination with 5.6% lactic acid, theoretically attenuating but not fully eliminating the noxious stimulus associated with lactic acid. 5.6% lactic acid was selected because it reliably induced sleep disturbances. Of note, effects of this concentration are surmountable by morphine and other NSAIDs in other assays modelling pain-suppressed behavior (80, 81, 115). Importantly, morphine within the dose range selected in the present study, as well as multiple NSAIDs are sufficient to attenuate stretching induced by lower doses of lactic acid (1.8%–3.2%) (79, 115). Secondly, morphine and AT-403 had divergent effects on NREM sleep regardless of lactic acid administration, and Figures 5J,L largely demonstrate a floor and ceiling effect following administration of 1 mg/kg morphine and 0.003 mg/kg AT-403, respectively. Together, these results suggest that analgesics, depending on their mechanism of action may have separate, and distinguishable effects on pain and sleep, and further studies are needed to understand distinct or synergistic effects of NOP agonists like AT-403 on pain and sleep in more long-lasting models of chronic pain.

In clinical settings, pain is primarily measured subjectively and is often referred to as “the fifth vital sign” given its status as an informative medical tool that has long-term consequences on health treatment and recovery. However, given that many pain treatments are associated with abuse potential, identifying objective ways to measure pain is of high priority in order to treat pain more responsibly. EEG is a valuable research tool to identify sleep patterns, neural oscillatory activity, and event-related potentials. Given that it can be recorded non-invasively in humans, it is a highly translational measure that could be used to identify possible biomarkers of pain, analgesia, and side effects using frequency analyses. Although previous studies reflect that acute pain in humans increases delta and gamma power oscillations (116–118), we did not find an effect of lactic acid on oscillatory activity during waking epochs (Supplementary Figure S3). Interestingly, morphine, meloxicam, and AT-403 produced disparate effects on qEEG profiles (Figure 6), likely attributed to their diverse mechanisms of action (43, 119). Importantly, qEEG can also be evaluated during NREM and REM sleep to evaluate sleep quality; increased NREM sleep delta power is often associated with deeper and better quality sleep (120, 121), whereas reduced delta power and increased sigma power is associated with light sleep in humans (121, 122). We found that NOP agonist AT-403, but not morphine, meloxicam, or lactic acid, increased delta and decreased sigma power during NREM sleep periods (Figures 6P,Q), indicating that AT-403 increased sleep depth, adding to the potential utility as an effective sleep-promoting agent.

When assessing the utility of a drug in a clinical setting it is important to consider potential side effects including abuse potential, cognitive and locomotor function, as well as effects on sleep. Although MOP agonists are highly efficacious analgesics, adverse effects are well documented and include high abuse potential, respiratory depression, tolerance, sedation, and sleep disruption (32, 33). NSAIDs, on the other hand, have mild gastrointestinal, cardiovascular, and renal side effects and minimal effects on sleep (43, 45–47) but are not as efficacious for severe pain (43, 44, 119). Previous literature has shown that NOP agonists are effective analgesics in acute (61–64) and chronic pain (61, 65, 66) models in animals, appear to have low abuse potential as they are not readily self-administered in rodents or monkeys (58, 59), and do not appear to produce physical dependence or tolerance (123, 124). Despite their largely favorable side effect profile, previous studies have shown that NOP agonists may impair locomotor function and/or induce sedation at high doses (60, 70, 114, 125). We found that AT-403 increased delta power during wake which is often associated with increased sleep drive, sedation, and/or decreased mental acuity and, at higher doses, decreased locomotor activity during EEG recording (Figures 3, 6). Additionally, the highest dose of AT-403 decreased motor performance in females on the Rotarod task, which measures balance and motor coordination (Figure 7). Although there were no direct sex differences, AT-403 did not impair locomotor function on the Rotarod task in males; these potential sex-differences may in part be attributed to non-significant but distinct differences in baseline motor performance (Figure 7). AT-403 also dose-dependently decreased REM sleep (Figures 3P, Q, 4G, 5F), which could result in negative effects on procedural memory consolidation, emotional regulation, and executive function that should be investigated in future studies (126, 127). Future studies (including drug optimization) may mitigate both concerns by developing NOP agonists with a broader dose-effect curve and therapeutic index that increase NREM sleep at doses that do not impact REM sleep or delta power while awake. Moreover, investigating chronopharmacology, or time of dosing may be considered. For example, NOP agonists could prove beneficial if specifically administered before bedtime to improve sleep and avoid daytime sedation.

While these studies begin to investigate the interactions between acute pain, analgesics and sleep, there are several limitations. First, lactic acid administration is a mildly noxious stimulus that does not influence quantitative EEG and has a short duration of action. We selected doses of all compounds/drugs tested, including lactic acid, based on their effects on sleep. Thus, 5.6% lactic acid was examined because lower doses did not reliably disrupt sleep (Figure 2). Similarly, we chose not to examine higher doses of morphine in combination with EEG because 1.0 mg/kg morphine disrupted sleep to a similar magnitude as higher doses (Figure 3), and we did not expect higher doses to improve sleep in the presence of LA (although we did not confirm this hypothesis). Second, combination EEG/behavioral experiments had lower sample sizes and only included male rats. Despite low samples sizes for correlative analyses between variables, morphine and AT-403 both produced potent sleep-altering effects perhaps inhibiting our ability to establish a clear relationship between sleep and antinociceptive activity (Figure 4). Future studies examining more potent pain stimuli and chronic pain models in both males and females are warranted to further investigate the impact of current and novel analgesics on indices of pain and sleep.

The development of novel analgesics that alleviate pain-induced sleep disruption is a crucial step in improving patient care, as sleep disturbances are prevalent in pain states, worsen pain sensitivity, and may persist despite efficacious doses of current analgesics. In the present study, we demonstrated (1) acute sleep disruptions following lactic acid administration, (2) NOP agonist, AT-403, promoted sleep in a pain-naïve and acute pain state, and (3) at the dose ranges examined, currently used analgesics morphine and meloxicam did not attenuate pain-induced behaviors nor improve sleep. While future studies are needed to investigate the bi-directional relationship between pain and sleep in more chronic pain models, these studies demonstrate that NOP agonists have promise for targeting pain-induced sleep disturbances, an understudied and undertreated symptom.

## Data Availability

The original contributions presented in the study are included in the article/Supplementary Material, further inquiries can be directed to the corresponding author.
