# Supplementary material for: NOP agonist AT-403 promoted sleep in lactic acid-induced acute pain model
Source: Front Pain Res (Lausanne). 2025 Sep 22;6:1659121. doi: 10.3389/fpain.2025.1659121 (PMC12497836; doi:10.3389/fpain.2025.1659121)
Supplement: Supplementary file 1 [file Datasheet1.pdf]

Supplemental Materials.

Subjects.

These studies comprised of six cohorts of rats (35 males, 20 females total). Cohort 1 (12 females) completed experiments 1a and 2 (n=8 females). Cohort 2 (12 males) completed experiments 1a and 2 (n=11 males). Cohort 3 completed experiments 1b (n=7 males), experiment 3 (meloxicam, n=9 males) and experiment 4 (n= 7 males). Cohort 4 (n=8 males) completed experiment 3 (morphine). Cohort 5 (n=15 males) completed experiment 3 (AT-403). Cohort 6 (n=8 males, 8 females) completed experiment 5.

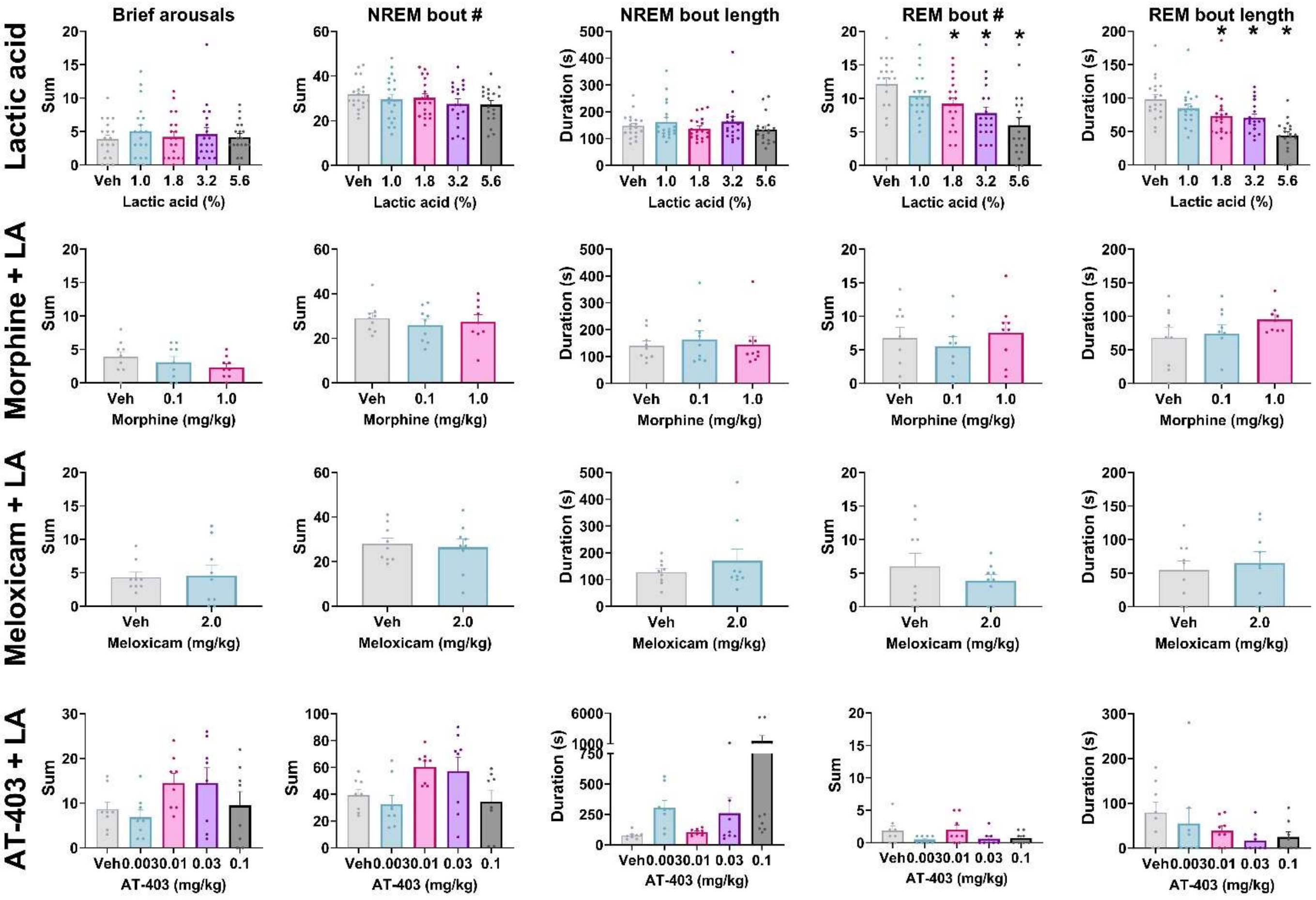

Supplemental Figure 1. Effects of lactic acid alone and in combination with analgesics on sleep continuity measures. Data were expressed as the summed frequency of brief arousals, NREM sleep bouts, REM sleep bouts, and the average duration of NREM and REM sleep bouts during the first 3 hours following administration of lactic acid (row 1), morphine + 5.6% lactic acid (row 2), meloxicam + 5.6% lactic acid (row 3), and AT-403 + 5.6% lactic acid (row 4). Data represent mean +/-SEM; circles represent individual animals. *$p<0.05$, significantly different from vehicle condition.

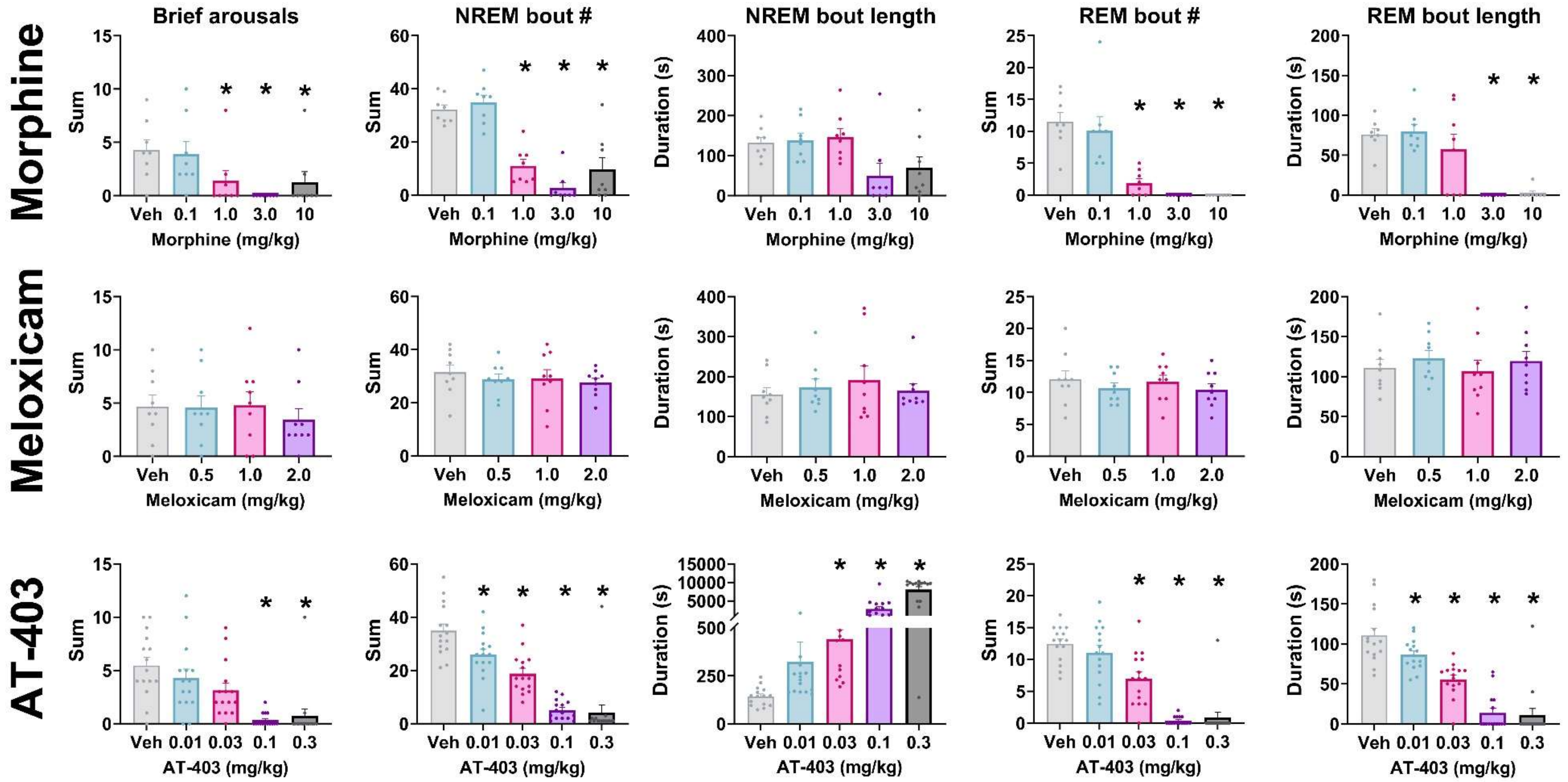

Supplemental Figure 2. Effects of morphine, meloxicam, and AT-403 on sleep continuity measures. Data were expressed as the summed frequency of brief arousals, NREM sleep bouts, REM sleep bouts, and the average duration of NREM and REM sleep bouts during the first 3 hours following administration of morphine (row 1), meloxicam (row 2), and AT-403 (row 3). Data represent mean +/-SEM; circles represent individual animals. *p<0.05, significantly different from vehicle condition.

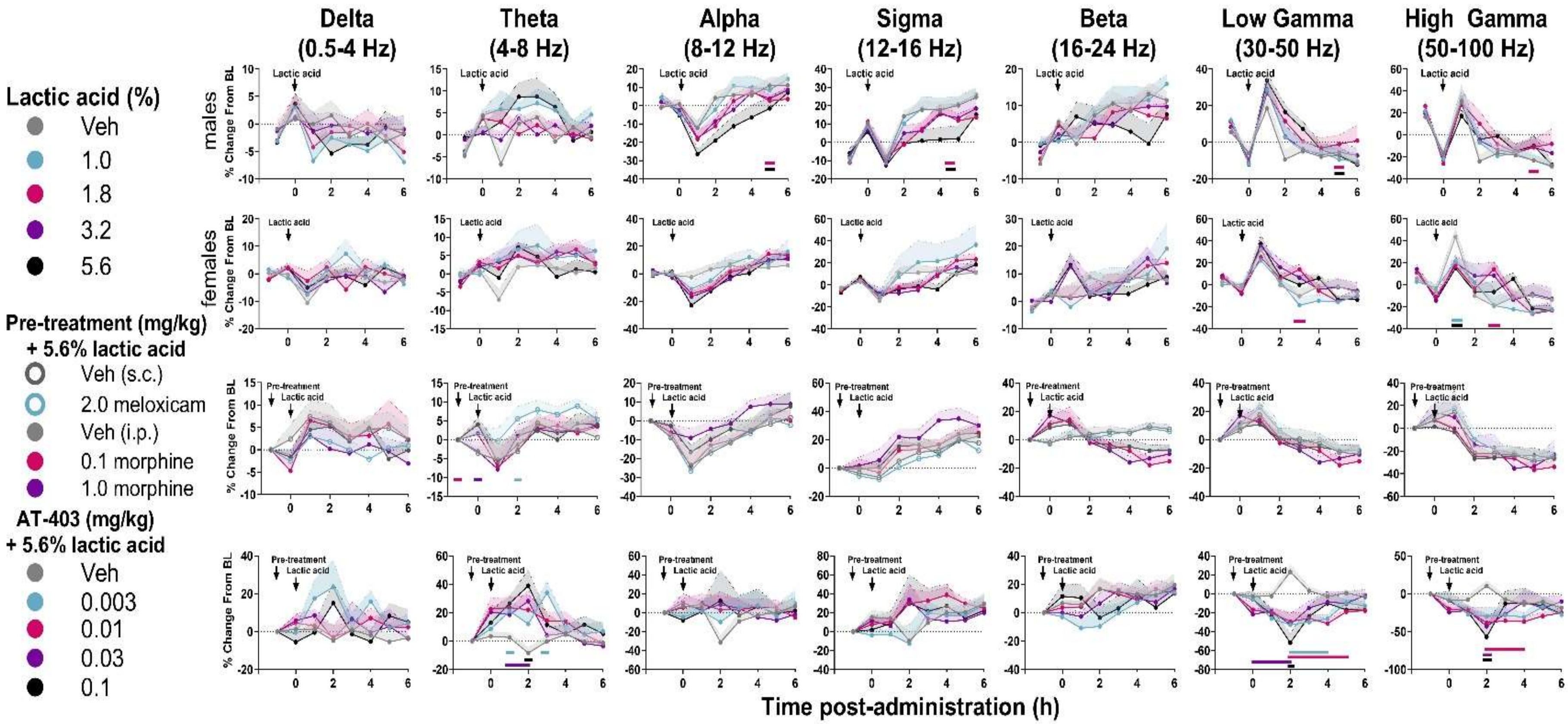

Supplemental Figure 3. Quantitative EEG activity following lactic acid and analgesic administration during wake. Relative power of frequency bands expressed as the % change of same-day BL during waking epochs following administration of lactic acid (row 1) or pre-treatment of morphine or meloxicam + 5.6% lactic acid (row 2), or AT-403 + 5.6% lactic acid (row 3). Data are expressed as mean +/-SEM. Colored horizontal lines signify doses significant from vehicle at those timepoints ($p<0.05$).

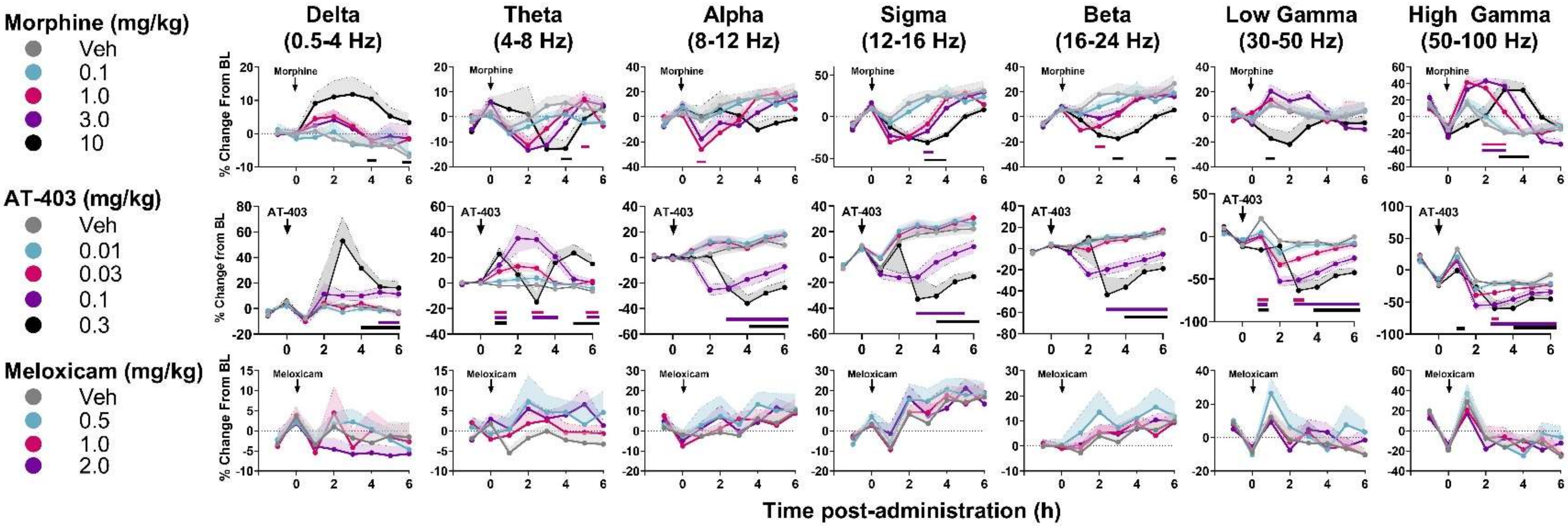

Supplemental Figure 4. Quantitative EEG activity following analgesic administration during wake. Relative power of frequency bands expressed as the % change of same-day BL during waking epochs following administration of morphine (row 1), AT-403 (row 2), and meloxicam (row 3). Data are expressed as mean +/-SEM. Colored horizontal lines signify doses significant from vehicle at those timepoints ($p<0.05$).

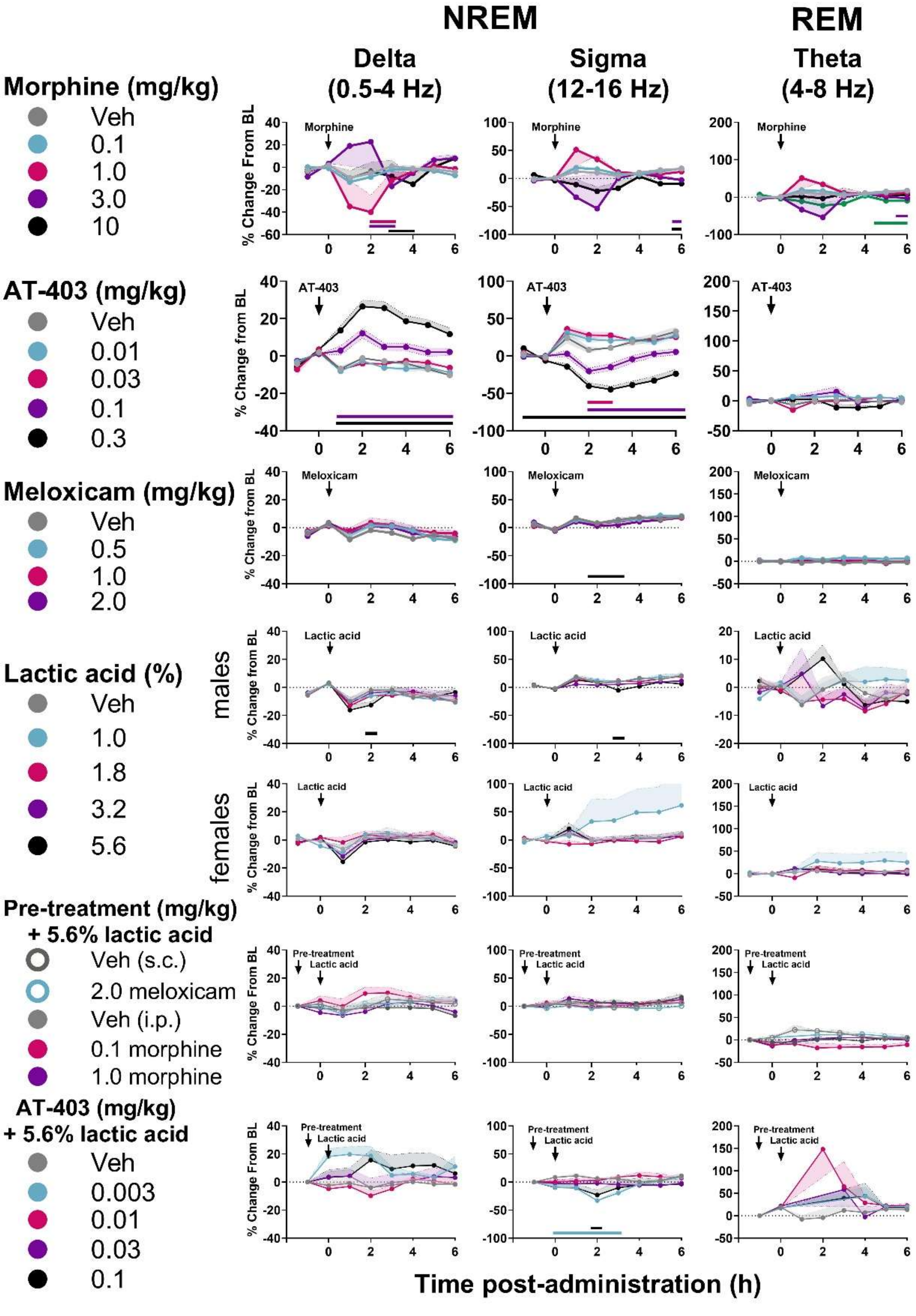

Supplemental Figure 5. Quantitative EEG activity after drug administration during NREM and REM sleep. Relative power of frequency bands expressed as the % change of same-day BL during NREM (column 1 and 2) and REM (column 3) sleep following administration of morphine, AT-403, meloxicam, lactic acid, or pre-treatment (morphine, meloxicam, or AT-403) + 5.6% lactic acid. Data represent mean +/-SEM. Colored horizontal lines signify doses significant from vehicle at those timepoints ($p<0.05$).

Supplemental Table 1. Effects of lactic acid and analgesics on sleep continuity							
	Factor	DF	F	P	*	*Post hoc* results	Significant Timepoints
**Lactic Acid**							
**Mixed effects one-way ANOVA**							
**Brief arousals**	Dose	3.368, 60.63	0.5936	0.6405	ns	N/A	
**NREM bout number**	Dose	3.276, 58.96	1.735	0.1652	ns	N/A	
**NREM bout duration**	Dose	2.590, 46.62	1.663	0.1933	ns	N/A	
**REM bout number**	Dose	2.807, 50.53	8.524	0.0002	***	1.8, 3.2, 5.6	0.0110, 0.0017, 0.0007
**REM bout duration**	Dose	2.598, 46.76	11.30	<0.0001	****	1.8, 3.2, 5.6	0.0110, 0.0031, 0.0003
**Morphine + 5.6% lactic acid**							
**Mixed effects one-way ANOVA**							
**Brief arousals**	Dose	4.529, 12.23	2.861	0.1049	ns	N/A	
**NREM bout number**	Dose	1.975, 15.80	0.8789	0.4333	ns	N/A	
**NREM bout duration**	Dose	1.852, 14.82	0.6709	0.5151	ns	N/A	
**REM bout number**	Dose	1.701, 13.61	1.0	0.3807	ns	N/A	
**REM bout duration**	Dose	1.503, 12.02	1.929	0.1909	ns	N/A	
**Meloxicam + 5.6% lactic acid**							
**Two-tailed paired t-test**							
	**Factor**	**DF**	**t**	**P**	*		
**Brief arousals**	Dose	8	0.1971	0.8487	ns	N/A	
**NREM bout number**	Dose	8	0.3455	0.7386	ns	N/A	
**NREM bout duration**	Dose	8	1.274	0.2386	ns	N/A	
**REM bout number**	Dose	8	1.222	0.2565	ns	N/A	
**REM bout duration**	Dose	8	0.7386	0.4812	ns	N/A	
**AT-403 + 5.6% lactic acid AT-403**							
**Mixed effects one-way ANOVA**							
	**Factor**	**DF**	**F**	**P**	*	***Post hoc* results**	**Significant Timepoints**
**Brief arousals**	Dose	2.822, 19.75	2.126	0.1321	ns	N/A	
**NREM bout number**	Dose	2.624, 18.37	3.511	0.0408	*	none	
**NREM bout duration**	Dose	1.042, 9.114	2.275	0.1653	ns	N/A	
**REM bout number**	Dose	2.065, 18.07	2.041	0.1578	ns	N/A	
**REM bout duration**	Dose	1.825, 12.77	1.740	0.2152	ns	N/A	

*p<0.05; ** p<0.01; *** p<0.001; ****p<0.0001, ns = not significant.

Supplemental Table 2. Effects of analgesics on sleep continuity							
**Mixed effects one-way ANOVA**							
	Factor	DF	F	P	*	*Post hoc* results	Significant Timepoints
**Morphine**							
**Brief arousals**	Dose	1.852, 12.96	5.467	0.0203	*	1, 3, 10	0.0219, 0.0116, 0.0222
**NREM bout number**	Dose	2.347, 16.43	26.51	<0.0001	****	1, 3, 10	0.0003, 0.0001, 0.0087
**NREM bout duration**	Dose	2.314, 16.20	4.241	0.0296	*	none	
**REM bout number**	Dose	1.468, 10.27	25.12	0.0002	***	1, 3, 10	0.0011, 0.0004, 0.0004
**REM bout duration**	Dose	1.601, 11.21	20.17	0.0003	***	3, 10	All <0.0001
**Meloxicam**							
**Brief arousals**	Dose	1.662, 13.30	0.6369	0.5164	ns	N/A	
**NREM bout number**	Dose	2.145, 17.16	0.6447	0.5475	ns	N/A	
**NREM bout duration**	Dose	1.609, 12.87	1.256	0.3082	ns	N/A	
**REM bout number**	Dose	2.305, 18.44	0.5878	0.5885	ns	N/A	
**REM bout duration**	Dose	2.223, 17.79	0.6015	0.5755	ns	N/A	
**AT-403**							
**Brief arousals**	Dose	3.203, 44.84	14.58	<0.0001	****	0.1, 0.3	<0.0001, 0.0002
**NREM bout number**	Dose	2.807, 39.29	42.50	<0.0001	****	0.01, 0.03, 0.1, 0.3	0.0197, 0.0022, <0.0001, <0.0001
**NREM bout duration**	Dose	1.891, 26.47	56.02	<0.0001	****	0.03, 0.1, 0.3	0.0002, 0.0020, <0.0001
**REM bout number**	Dose	2.841, 39.77	47.53	<0.0001	****	0.03, 0.1, 0.3	0.0008, <0.0001, <0.0001
**REM bout duration**	Dose	5.153, 30.14	44.23	<0.0001	****	0.01, 0.03, 0.1, 0.3	0.0325, 0.0003, <0.0001, <0.0001

*p<0.05; ** p<0.01; *** p<0.001; ****p<0.0001, ns = not significant.

Supplemental Table 3. Effects of lactic acid on quantitative EEG in male rats

Mixed effects two-way ANOVA

	**Factor**	**DF**	**F**	**p**	*****	***Post hoc* results**	**Significant Timepoints**
Delta (Wake)	Concentration	3.033,30.33	0.9162	0.4456	ns	N/A	
	Time	1.595,15.95	0.2148	0.7598	ns		
	Interaction	5.592,55.92	0.2796	0.3040	ns		
Theta (Wake)	Concentration	2.214,21.24	1.606	0.2235	ns	N/A	
	Time	2.001,20.01	2.182	0.1389	ns		
	Interaction	3.887,38.87	1.651	0.1824	ns		
Alpha (Wake)	Concentration	2.680,26.80	4.312	0.0158	*	1%	none
	Time	3.012,30.12	35.04	<0.0001	****	1.8%	4
	Interaction	6.108,61.08	1.269	0.2844	ns	3.2%	none
						5.6%	4
Sigma (Wake)	Concentration	2.308,23.08	2.627	0.0871	ns	1%	none
	Time	3.359,33.59	25.70	<0.0001	****	1.8%	4
	Interaction	6.367,63.67	0.9486	0.4706	ns	3.2%	none
						5.6%	4, 6
Beta (Wake)	Concentration	1.948,19.48	0.6311	0.5386	ns	1%	none
	Time	2.757,27.57	4.492	0.0126	*	1.8%	none
	Interaction	6.102,61.01	1.609	0.1590	ns	3.2%	none
						5.6%	none
Low Gamma (Wake)	Concentration	2.627,26.27	1.379	0.2714	ns	1%	none
	Time	3.096,30.96	41.72	<0.0001	****	1.8%	4
	Interaction	6.943,69.43	0.3471	0.1586	ns	3.2%	none
						5.6%	4
High Gamma (Wake)	Concentration	2.103,21.03	0.9870	0.3930	ns	1%	none
	Time	3.482,34.82	33.97	<0.0001	****	1.8%	4
	Interaction	6.708,67.08	1.303	0.2642	ns	3.2%	none
						5.6%	none
Sigma (NREM)	Concentration	2.838,28.38	2.628	0.0723	ns	1%	none
	Time	2.072,20.72	6.472	0.0061	**	1.8%	none
	Interaction	5.945,58.55	1.655	0.1490	ns	3.2%	none
						5.6%	none
Delta (NREM)	Concentration	3.184,31.84	0.5295	0.6756	ns	1%	none
	Time	2.254,22.54	8.624	0.0012	**	1.8%	none
	Interaction	4.973,48.99	2.345	0.0553	ns	3.2%	none
						5.6%	4
Locomotor activity	Concentration	2.654, 26.54	0.6950	0.5467	ns	1%	none
	Time	3.433, 34.33	60.89	<0.0001	****	1.8%	none
	Interaction	6.569, 65.69	1.361	0.2395	ns	3.2%	none
						5.6%	none

*p<0.05; ** p<0.01; *** p<0.001; ****p<0.0001, ns = not significant

Supplemental Table 4. Effects of lactic acid on quantitative EEG in female rats							
Mixed effects two-way ANOVA							
	Factor	DF	F	p	*	*Post hoc* results	Significant Timepoints
Delta (Wake)	Concentration	2.65,18.55	0.8709	0.4622	ns	1%	none
	Time	2.27,15.91	4.626	0.0227	*	1.8%	none
	Interaction	4.21,29.46	1.406	0.2553	ns	3.2%	none
						5.6%	none
Theta (Wake)	Concentration	1.87,13.08	1.489	0.2606	ns	N/A	
	Time	2.66,18.60	3.013	0.0613	ns	N/A	
	Interaction	3.84,26.09	0.9703	0.4374	ns	N/A	
Alpha (Wake)	Concentration	2.43,17.01	1.180	0.3393	ns	1%	none
	Time	2.55,17.87	19.92	<0.0001	****	1.8%	none
	Interaction	4.94, 4.60	1.649	0.1740	ns	3.2%	none
						5.6%	none
Sigma (Wake)	Concentration	1.20, 8.83	0.2993	0.4368	ns	1%	none
	Time	1.76,12.31	9.748	0.0036	**	1.8%	none
	Interaction	2.40,16.83	0.0859	0.2680	ns	3.2%	none
						5.6%	none
Beta (Wake)	Concentration	1.57,10.96	0.559	0.9103	ns	1%	none
	Time	1.97,13.77	8.110	0.0048	**	1.8%	none
	Interaction	3.81, 26.7	1.626	0.1987	ns	3.2%	none
						5.6%	none
Low Gamma (Wake)	Concentration	2.86,20.02	1.989	0.1501	ns	1%	none
	Time	3.47,24.27	30.39	<0.0001	****	1.8%	5
	Interaction	5.72,40.03	1.815	0.1236	ns	3.2%	none
						5.6%	none
High Gamma (Wake)	Concentration	2.39,16.75	0.60	0.4982	ns	1%	3
	Time	3.28,22.99	19.13	<0.0001	****	1.8%	5
	Interaction	4.93,34.52	0.18	0.0920	ns	3.2%	none
						5.6%	3
Sigma (NREM)	Concentration	1.09, 7.60	1.027	0.3498	ns	N/A	
	Time	1.25, 8.75	2.651	0.1362	ns	N/A	
	Interaction	1.23, 8.84	1.177	0.3233	ns	N/A	
Delta (NREM)	Concentration	1.52, 10.6	0.7072	0.4769	ns	1%	none
	Time	2.87, 20.11	12.73	<0.0001	****	1.8%	none
	Interaction	4.01,27.35	1.225	0.3232	ns	3.2%	none
						5.6%	none
Locomotor activity	Concentration	2.729, 29.72	2.377	0.162	ns	1%	none
	Time	2.999, 21.00	33.27	<0.0001	****	1.8%	none
	Interaction	4.245, 29.71	1.671	0.1804	ns	3.2%	none
						5.6%	none
*p<0.05; ** p<0.01; *** p<0.001; ****p<0.0001, ns = not significant							

Supplemental Table 5. Effects of morphine on quantitative EEG in male rats (first 6 hours)							
Repeated measures 2-way ANOVA's							
	Factor	DF	F	p	*	*Post hoc* results	Significant Timepoints
Delta (Wake)	Dose	1.718,12.02	9.579	0.0042	**	0.1 mg/kg	none
	Time	2.962,20.73	6.903	0.0022	**	1 mg/kg	none
	Interaction	4.675,32.72	1.141	0.3575	ns	3 mg/kg	none
						10 mg/kg	6, 8
Theta (Wake)	Dose	1.352,9.462	5.802	0.0310	*	0.1 mg/kg	none
	Time	3.080,21.56	1.271	0.3096	ns	1 mg/kg	7
	Interaction	2.895,20.27	4.375	0.0166	*	3 mg/kg	none
						10 mg/kg	6
Alpha (Wake)	Dose	1.427,9.988	4.311	0.0545	ns	0.1 mg/kg	none
	Time	2.000,14.00	1.667	0.2241	ns	1 mg/kg	3
	Interaction	4.364,30.55	4.118	0.0074	**	3 mg/kg	none
						10 mg/kg	none
Sigma (Wake)	Dose	1.511,10.58	34.51	<0.0001	****	0.1 mg/kg	none
	Time	2.391,16.74	8.191	0.0023	**	1 mg/kg	none
	Interaction	3.628,25.39	7.274	0.0006	***	3 mg/kg	5
						10 mg/kg	5, 6
Beta (Wake)	Dose	1.210,8.469	9.575	0.0114	*	0.1 mg/kg	none
	Time	2.776,19.43	5.852	0.0059	**	1 mg/kg	4
	Interaction	3.523,24.66	2.141	0.1125	ns	3 mg/kg	none
						10 mg/kg	5, 8
Low Gamma (Wake)	Dose	1.541,10.79	2.023	0.1829	ns	0.1 mg/kg	none
	Time	2.799,19.59	2.410	0.1010	ns	1 mg/kg	none
	Interaction	3.608,25.26	3.458	0.0251	*	3 mg/kg	none
						10 mg/kg	3
High Gamma (Wake)	Dose	1.967,13.77	35.04	<0.0001	****	0.1 mg/kg	none
	Time	2.971,20.80	3.128	0.0480	*	1 mg/kg	4, 5
	Interaction	4.671,32.70	11.82	<0.0001	****	3 mg/kg	4, 5
						10 mg/kg	5, 6
Sigma (NREM)	Dose	5, 35	6.303	0.0003	***	0.1 mg/kg	none
	Time	5, 35	7.495	<0.0001	****	1 mg/kg	none
	Interaction	25, 117	4.626	<0.0001	****	3 mg/kg	8
						10 mg/kg	8
Delta (NREM)	Dose	5, 35	7.8	<0.0001	****	0.1 mg/kg	none
	Time	7, 49	5.495	0.0001	***	1 mg/kg	none
	Interaction	35, 186	4.212	<0.0001	****	3 mg/kg	8
						10 mg/kg	8
Locomotor activity (sum)	Dose	1.609, 11.26	3.303	0.0818	ns	0.1 mg/kg	1
	Time	3.687, 25.81	23.08	<0.000`	****	1 mg/kg	2, 3, 6, 8
	Interaction	3.467, 24.27	4.077	0.0144	*	3 mg/kg	4, 10,
						10 mg/kg	4, 8, 9
*p<0.05; ** p<0.01; *** p<0.001; ****p<0.0001, ns = not significant							

Supplemental Table 6. Effects of meloxicam on quantitative EEG in male rats							
**Mixed effects two-way ANOVA**							
	**Factor**	**DF**	**F**	**p**	*****	***Post hoc* results**	**Significant Timepoints**
Delta (Wake)	Dose	1.943,15.55	0.7104	0.5029	ns		
	Time	1.606,12.85	0.7720	0.4555	ns		
	Interaction	3.521,28.17	1.110	0.3672	ns		
Theta (Wake)	Dose	1.439,11.51	1.447	0.2669	ns		
	Time	2.481,19.85	1.774	0.1905	ns		
	Interaction	3.959,31.68	0.6242	0.6471	ns		
Alpha (Wake)	Dose	1.435,11.48	0.3315	0.6546	ns		
	Time	1.714,13.71	2.066	0.1679	ns		
	Interaction	4.570,36.56	0.7674	0.5689	ns		
Sigma (Wake)	Dose	1.839,14.72	0.2944	0.7315	ns	0.5 mg/kg	none
	Time	2.335,18.68	12.20	0.0003	***	1 mg/kg	none
	Interaction	4.896,39.17	0.7332	0.6003	ns	2 mg/kg	none
Beta (Wake)	Dose	1.397,11.18	0.6261	0.4973	ns	0.5 mg/kg	none
	Time	2.038,16.30	5.095	0.0187	*	1 mg/kg	none
	Interaction	4.083,32.66	0.5890	0.6762	ns	2 mg/kg	none
Low Gamma (Wake)	Dose	2.065,16.52	0.5915	0.5700	ns	0.5 mg/kg	none
	Time	2.524,20.19	7.304	0.0025	**	1 mg/kg	none
	Interaction	5.215,41.71	1.851	0.1212	ns	2 mg/kg	none
High Gamma (Wake)	Dose	2.058,16.47	0.1390	0.8766	ns	0.5 mg/kg	none
	Time	3.001,24.01	11.20	<0.0001	****	1 mg/kg	none
	Interaction	4.049,32.39	1.151	0.3508	ns	2 mg/kg	none
Sigma (NREM)	Dose	1.834,14.67	0.8752	0.4288	ns	0.5 mg/kg	none
	Time	1.815,14.52	8.465	0.0044	**	1 mg/kg	none
	Interaction	4.939,39.51	0.7086	0.6189	ns	2 mg/kg	5, 6
Delta (NREM)	Dose	1.771,14.17	0.9049	0.4152	ns	0.5 mg/kg	none
	Time	2.310,18.48	11.87	0.0003	***	1 mg/kg	none
	Interaction	4.254,34.03	1.054	0.3966	ns	2 mg/kg	none
Locomotor activity	Dose	2.117, 16.94	1.376	0.2804	ns	0.5 mg/kg	none
	Time	3.537, 28.29	34.15	<0.0001	****	1 mg/kg	none
	Interaction	5.188, 41.51	0.6787	0.6472	ns	2 mg/kg	none
*p<0.05; ** p<0.01; *** p<0.001; ****p<0.0001, ns = not significant							

Supplemental Table 7. Effects of AT-403 on quantitative EEG in male rats							
Repeated measures 2-way ANOVA's							
	Factor	DF	F	p	*	*Post hoc* results	Significant Timepoints
Delta (Wake)	Dose	2.683, 37.56	13.80	<0.0001	****	0.01 mg/kg	none
	Time	2.868, 40.15	13.36	<0.0001	****	0.03 mg/kg	14
	Interaction	5.467, 74.11	4.894	0.0004	***	0.10 mg/kg	7-12, 24
						0.30 mg/kg	6-13
Theta (Wake)	Dose	2.961, 41.46	8.97	0.0001	***	0.01 mg/kg	none
	Time	3.060, 42.84	15.93	<0.0001	****	0.03 mg/kg	3, 5, 8, 23
	Interaction	6.625, 89.80	8.163	<0.0001	****	0.10 mg/kg	3, 5, 6, 8
						0.30 mg/kg	3, 7-11, 13, 14
Alpha (Wake)	Dose	3.058, 42.82	15.56	<0.0001	****	0.01 mg/kg	none
	Time	1.786, 25	1.683	0.2077	ns	0.03 mg/kg	none
	Interaction	7.979, 108.2	7.521	<0.0001	****	0.10 mg/kg	5-9
						0.30 mg/kg	6-12
Sigma (Wake)	Dose	2.910, 40.74	13.98	<0.0001	****	0.01 mg/kg	none
	Time	3.830, 53.62	17.80	<0.0001	****	0.03 mg/kg	None
	Interaction	7.292, 98.84	6.197	<0.0001	****	0.10 mg/kg	5-7, 9
						0.30 mg/kg	6-10, 21
Beta (Wake)	Dose	2.749, 38.49	11.57	<0.0001	****	0.01 mg/kg	none
	Time	2.389, 33.45	9.363	0.0003	***	0.03 mg/kg	none
	Interaction	5.827, 78.98	10.15	<0.0001	****	0.10 mg/kg	5-9
						0.30 mg/kg	6-11
Low Gamma (Wake)	Dose	3.276, 45.86	11.01	<0.0001	****	0.01 mg/kg	none
	Time	6.082, 85.15	58.93	<0.0001	****	0.03 mg/kg	3, 5, 16,
	Interaction	8.699, 117.9	5.938	<0.0001	****	0.10 mg/kg	3, 5-8, 11
						0.30 mg/kg	3, 6-14, 23
High Gamma (Wake)	Dose	3.294, 46.11	7.383	0.0003	***	0.01 mg/kg	none
	Time	6.054, 84.76	60.24	<0.0001	****	0.03 mg/kg	5, 16
	Interaction	9.577, 129.8	2.674	0.0059	**	0.10 mg/kg	5-8, 13, 24
						0.30 mg/kg	3, 6-14, 23
Sigma (NREM)	Dose	2.349, 32.88	21.50	<0.0001	****	0.01 mg/kg	none
	Time	4.920, 68.87	32.13	<0.0001	****	0.03 mg/kg	4, 5,
	Interaction	5.742, 72.65	4.473	0.0008	***	0.10 mg/kg	4-12, 23
						0.30 mg/kg	1-17, 21-23
Delta (NREM)	Dose	1.964, 27.49	34.49	<0.0001	****	0.01 mg/kg	none
	Time	4.850, 67.90	27.98	<0.0001	****	0.03 mg/kg	10-13
	Interaction	7.089, 89.69	5.464	<0.0001	****	0.10 mg/kg	3-13, 23
						0.30 mg/kg	3-17, 21-23
Locomotor activity	Dose	2.654, 74.85	2.785	0.0604	ns	0.01 mg/kg	21
	Time	5.346, 74.85	47.27	<0.0001	****	0.03 mg/kg	3, 16
	Interaction	9.800, 137.2	2.071	0.0318	*	0.10 mg/kg	3, 18
						0.30 mg/kg	4, 5, 20, 21
*p<0.05; ** p<0.01; *** p<0.001; ****p<0.0001, ns = not significant							

Supplemental Table 8. Effects of analgesics on sleep in male rats							
Repeated measures one-way ANOVA							
Morphine							
	Factor	DF	F	P	*	Significant doses	P
Wake (1 hour sum)	Dose	7	13.50	0.0003	***	1, 3 mg/kg	0.0054, 0.0115
NREM (1 hour sum)	Dose	7	13.22	0.0007	***	1, 3 mg/kg	0.0057, 0.0038
REM (1 hour sum)	Dose	7	4.137	0.0399	*	none	
Wake (6 hour sum)	Dose	7	53.48	<0.0001	****	1, 3, 10 mg/kg	0.0075, 0.0015, <0.0001
NREM (6 hour sum)	Dose	7	31.12	<0.0001	****	3, 10 mg/kg	0.0069, 0.0008
REM (6 hour sum)	Dose	7	41.02	<0.0001	****	1, 3, 10 mg/kg	0.0390, 0.0028, 0.0001
Meloxicam							
Wake (1 hour Sum)	Dose	8	0.6290	0.5696	ns	N/A	
NREM (1 hour Sum)	Dose	8	0.9560	0.4110	ns	N/A	
REM (1 hour Sum)	Dose	8	0.2613	0.8176	ns	N/A	
Wake (6 hour sum)	Dose	8	4.337	0.0244	*	2 mg/kg	0.0229
NREM (6 hour sum)	Dose	8	2.406	0.1033	ns	N/A	
REM (6 hour sum)	Dose	8	3.212	0.0700	ns	N/A	
AT-403							
Wake (1 hour sum)	Dose	4, 14	-65.75	>0.9999	ns	0.01, 0.03, 0.10, 0.30	0.002, <0.0001, <0.0001,<0.0001
NREM (1 hour sum)	Dose	4, 14	-88.09	>0.9999	ns	0.01, 0.03, 0.10, 0.30	0.0025, <0.0001, <0.0001,
REM (1 hour sum)	Dose	4, 14	6.862	0.0025	**	0.03, 0.10	0.0049, 0.0060
Wake (6 hour sum)	Dose	4, 14	-33.15	>0.9999	ns	0.01, 0.03, 0.10, 0.30	0.0042, 0.0005, <0.0001, <0.0001
NREM (6 hour sum)	Dose	4, 14	-9.405	>0.9999	ns	0.01, 0.03, 0.10, 0.30	0.0004, <0.0001, <0.0001, <0.0001
REM (6 hour sum)	Dose	4, 14	458.7	<0.0001	****	0.03, 0.10, 0.30	<0.0001, <0.0001, <0.0001
Morphine + 5.6% lactic acid							
Wake (1 hour Sum)	Dose	1.559, 12.47	1.346	0.2875	ns	N/A	
NREM (1 hour Sum)	Dose	1.618, 12.95	1.256	0.3083	ns	N/A	
REM (1 hour Sum)	Dose	1.418, 11.35	1.249	0.3073	ns	N/A	
Wake (6 hour sum)	Dose	1.666, 13.32	0.3264	0.6887	ns	N/A	
NREM (6 hour sum)	Dose	1.636, 13.09	0.2717	0.7231	ns	N/A	
REM (6 hour sum)	Dose	1.937, 15.49	2.814	0.0920	ns	N/A	
Meloxicam + 5.6% lactic acid							
Two-tailed paired t-test							
	Factor	DF	t	P	*		
Wake (1 hour) Sum)	Dose	8	1.404	0.1979	ns		
NREM (1 hour)	Dose	8	1.328	0.2209	ns		
REM (1 hour) Sum)	Dose	8	1.964	0.0851	ns		
Wake (6 hour) sum)	Dose	8	0.3014	0.7708	ns		
NREM (6 hour)	Dose	8	0.7016	0.5028	ns		
REM (6 hour) sum)	Dose	8	0.3785	0.7149	ns		
AT-403 + 5.6% lactic acid							
Repeated measures one-way ANOVA							
	Factor	DF	F	P	*	Significant doses	P
Wake (1 hour Sum)	Dose	2.481, 34.74	63.82	<0.0001	****	0.003, 0.01, 0.03, 0.1	0.0020, <0.0001, <0.0001, <0.0001
NREM (1 hour Sum)	Dose	2.495, 34.93	68.99	<0.0001	****	0.003, 0.01, 0.03, 0.1	0.0025, <0.0001, <0.0001, <0.0001
REM (1 hour Sum)	Dose	2.280, 31.92	6.713	0.0026	**	0.03, 0.10	0.0049, 0.0060
Wake (6 hour sum)	Dose	3.042, 42.59	44.06	<0.0001	****	0.003, 0.01, 0.03, 0.1	0.0042, 0.0005, <0.0001, 0.0001
NREM (6 hour sum)	Dose	2.676, 37.47	82.59	<0.0001	****	0.003, 0.01, 0.03, 0.1	0.0004, <0.0001, <0.0001, <0.0001
REM (6 hour sum)	Dose	2.467, 34.54	72.97	<0.0001	****	0.01, 0.03, 0.1	<0.0001, <0.0001, <0.0001
*p<0.05; ** p<0.01; *** p<0.001; ****p<0.0001, ns = not significant							

Supplemental Table 9. Effects of morphine + 5.6% lactic acid on quantitative EEG in male rats							
Mixed effects two-way ANOVA							
	**Factor**	**DF**	**F**	**p**	*****	***Post hoc***	**Significant Timepoints**
Delta (Wake)	Dose	1.163, 9.302	0.6490	0.4635	ns	N/A	
	Time	2.514, 20.11	2.458	0.1008	ns	N/A	
	Interaction	2.080, 16.64	0.8817	0.4364	ns	N/A	
Theta (Wake)	Dose	1.422, 11.38	0.06590	0.8803	ns	0.5 mg/kg	1
	Time	2.695, 21.56	3.849	0.0271	*	1 mg/kg	2
	Interaction	4.185, 33.48	0.9117	0.4719	ns		
Alpha (Wake)	Dose	1.668, 13.34	3.403	0.0706	ns	0.5 mg/kg	none
	Time	1.812, 14,49	6.532	0.0109	*	1 mg/kg	none
	Interaction	4.409, 35.27	1.084	0.3825	ns		
Sigma (Wake)	Dose	1.525, 12.20	2.326	0.1465	ns	0.5 mg/kg	none
	Time	2.785, 22,28	14.49	<0.0001	****	1 mg/kg	none
	Interaction	4.943, 39.54	1.128	0.3612	ns		
Beta (Wake)	Dose	2.240, 17.92	13.80	0.0002	***	0.5 mg/kg	none
	Time	1.795, 14.36	0.1367	0.8524	ns	1 mg/kg	none
	Interaction	4.379, 35.04	0.9815	0.4354	ns		
Low Gamma (Wake)	Dose	1.795, 14.36	0.1367	0.8524	ns	0.5 mg/kg	none
	Time	2.240, 17.92	13.80	0.0002	***	1 mg/kg	none
	Interaction	4.379, 35.03	0.9815	0.4354	ns		
High Gamma (Wake)	Dose	1.221, 9.770	0.3889	0.5884	ns	0.5 mg/kg	none
	Time	2.639, 21.11	17.48	<0.0001	****	1 mg/kg	none
	Interaction	3,720, 29.76	0.8879	0.4771	ns		
Sigma (NREM)	Dose	1.325, 10.60	0.5159	0.5384	ns	0.5 mg/kg	none
	Time	2.633, 21.07	3.411	0.411	*	1 mg/kg	none
	Interaction	3.149, 22.94	1.307	0.2966	ns		
Delta (NREM)	Dose	1.313, 10.50	2.485	0.1409	ns	N/A	
	Time	2.482, 19.87	2.631	0.0872	ns	N/A	
	Interaction	3.597, 26.21	1.939	0.1391	ns	N/A	
Locomotor activity	Dose	1.755, 21.01	31.35	<0.0001	****	0.5 mg/kg	none
	Time	1.755, 14.04	1.121	0.3459	ns	1 mg/kg	none
	Interaction	4.704, 37.63	0.5121	0.7550	ns		
*p<0.05; ** p<0.01; *** p<0.001; ****p<0.0001, ns = not significant							

Supplemental Table 10. Effects of meloxicam + 5.6% lactic acid on quantitative EEG in male rats						
Mixed effects two-way ANOVA						
	Factor	DF	F	p	*	Significant Timepoints
Delta (Wake)	Vehicle + 5.6% LA vs. 2 mg/kg + 5.6% LA	1, 8	1.792	0.2174	ns	
	Time	7, 56	0.8480	0.5528	ns	
	Interaction	7, 56	0.7385	0.6403	ns	
Theta (Wake)	Vehicle + 5.6% LA vs. 2 mg/kg + 5.6% LA	1, 8	11.52	0.0094	**	4
	Time	7, 56	3.421	0.0041	**	
	Interaction	7, 56	0.8591	0.5442	ns	
Alpha (Wake)	Vehicle + 5.6% LA vs. 2 mg/kg + 5.6% LA	1, 8	0.007580	0.9328	ns	none
	Time	7, 56	10.19	<0.0001	****	
	Interaction	7, 56	0.3058	0.9484	ns	
Sigma (Wake)	Vehicle + 5.6% LA vs. 2 mg/kg + 5.6% LA	1, 8	0.1768	0.6852	ns	none
	Time	7, 56	9.007	<0.0001	****	
	Interaction	7, 56	0.1637	0.9913	ns	
Beta (Wake)	Vehicle + 5.6% LA vs. 2 mg/kg + 5.6% LA	1, 8	0.03046	0.8658	ns	none
	Time	7, 56	3.160	0.0069	**	
	Interaction	7, 56	0.3395	0.9323	ns	
Low Gamma (Wake)	Vehicle + 5.6% LA vs. 2 mg/kg + 5.6% LA	1, 8	0.5447	0.4816	ns	none
	Time	7, 56	8.158	<0.0001	****	
	Interaction	7, 56	0.2338	0.9753	ns	
High Gamma (Wake)	Vehicle + 5.6% LA vs. 2 mg/kg + 5.6% LA	1, 8	0.3356	0.5783	ns	none
	Time	7, 56	10.29	<0.0001	****	
	Interaction	7, 56	0.1733	0.9897	ns	
Sigma (NREM)	Vehicle + 5.6% LA vs. 2 mg/kg + 5.6% LA	1, 8	2.005	0.1945	ns	
	Time	7, 56	1.107	0.3717	ns	
	Interaction	7, 56	0.5581	0.7862	ns	
Delta (NREM)	Vehicle + 5.6% LA vs. 2 mg/kg + 5.6% LA	1, 8	0.006669	0.9369	ns	
	Time	7, 56	1.950	0.0787	ns	
	Interaction	7, 56	0.6532	0.7100	ns	
Locomotor activity	Vehicle + 5.6% LA vs. 2 mg/kg + 5.6% LA	1, 8	1.744	0.2231	ns	none
	Time	2.904,	26.11	<0.0001	****	
	Interaction	3.3,	1.137	0.3553	ns	
*p<0.05; ** p<0.01; *** p<0.001; ****p<0.0001, ns = not significant						

Supplemental Table 11. Effects of AT-403 + 5.6% lactic acid on quantitative EEG in male rats							
Mixed effects two-way ANOVA							
	**Factor**	**DF**	**F**	**p**	*****	***Post hoc* results**	**Significant Timepoints**
Delta (Wake)	Dose	2.454, 17.18	1.917	0.1716	ns	N/A	
	Time	2.280, 15.96	0.9865	0.4045	ns		
	Interaction	3.111, 21	1.810	0.1750	ns		
Theta (Wake)	Dose	1.779, 12.46	2.068	0.1702	*	0.003 mg/kg	3, 5
	Time	2.951, 20.66	8.286	0.0009	***	0.01 mg/kg	None
	Interaction	3.220, 21.74	3.240	0.0391	*	0.03 mg/kg	3, 4
						0.10 mg/kg	4
Alpha (Wake)	Dose	1.455, 10.19	0.0538	0.9003	ns	N/A	
	Time	1.758, 12.31	0.5382	0.5749	ns		
	Interaction	1.957, 13.21	2.113	0.1605	ns		
Sigma (Wake)	Dose	1.557, 10.90	0.4442	0.6048	ns	N/A	
	Time	1.529, 10.70	3.498	0.0767	ns		
	Interaction	1.835, 12.38	2.333	0.1407	ns		
Beta (Wake)	Dose	2.086, 14.60	1.163	0.3420	ns	0.003 mg/kg	10
	Time	2.249, 15.74	4.669	0.0225	*	0.01 mg/kg	none
	Interaction	4.789, 32.33	1.862	0.1310	ns	0.03 mg/kg	none
						0.10 mg/kg	none
Low Gamma (Wake)	Dose	2.089, 14.62	3.971	0.0405	*	0.003 mg/kg	4-6
	Time	2.587, 18.11	4.446	0.0199	*	0.01 mg/kg	4-7
	Interaction	4.179, 28.21	2.961	0.0351	*	0.03 mg/kg	2-4
						0.10 mg/kg	4
High Gamma (Wake)	Dose	2.309, 16.16	1.449	0.2650	ns	0.003 mg/kg	none
	Time	2.410, 16.87	4.817	0.0177	*	0.01 mg/kg	4-6
	Interaction	3.845, 25.95	2.139	0.1066	ns	0.03 mg/kg	4
						0.10 mg/kg	4
Sigma (NREM)	Dose	1.669, 11.68	2.388	0.1402	ns	0.003 mg/kg	2-5
	Time	2.419, 16.93	4.754	0.0183	*	0.01 mg/kg	none
	Interaction	3.028, 20.94	2.205	0.1172	ns	0.03 mg/kg	8
						0.10 mg/kg	4
Delta (NREM)	Dose	1.861, 13.03	3.080	0.0830	ns	N/A	
	Time	2.133, 14.93	0.8359	0.4597	ns		
	Interaction	2.943, 20.36	2.099	0.1328	ns		
Locomotor activity	Dose	4.607, 32.25	17.66	<0.0001	****	0.003 mg/kg	2-4
	Time	2.731, 19.12	2.512	0.0936	ns	0.01 mg/kg	4
	Interaction	5.872, 41.11	3.284	0.0103	*	0.03 mg/kg	2-4
						0.10 mg/kg	2-4
*p<0.05; ** p<0.01; *** p<0.001; ****p<0.0001, ns = not significant							
